# Microglia Polarization, Gene-Environment Interactions and Wnt/β-Catenin Signaling: Emerging Roles of Glia-Neuron and Glia-Stem/Neuroprogenitor Crosstalk for Dopaminergic Neurorestoration in Aged Parkinsonian Brain

**DOI:** 10.3389/fnagi.2018.00012

**Published:** 2018-02-12

**Authors:** Francesca L'Episcopo, Cataldo Tirolo, Maria F. Serapide, Salvatore Caniglia, Nunzio Testa, Loredana Leggio, Silvia Vivarelli, Nunzio Iraci, Stefano Pluchino, Bianca Marchetti

**Affiliations:** ^1^Oasi ResearchInstitute-IRCCS, Troina, Italy; ^2^Department of Biomedical and Biotechnological Sciences, Medical School, University of Catania, Catania, Italy; ^3^Division of Stem Cell Neurobiology, Department of Clinical Neurosciences, Wellcome Trust-Medical Research Council Stem Cell Institute, NIHR Biomedical Research Centre, University of Cambridge, Cambridge, United Kingdom

**Keywords:** Parkinson's disease, neuroinflammation, Wnt/β-catenin signaling, aging, dopaminergic neurons, neurogenesis, neurodegeneration, neuroprotection

## Abstract

Neuroinflammatory processes are recognized key contributory factors in Parkinson's disease (PD) physiopathology. While the causes responsible for the progressive loss of midbrain dopaminergic (mDA) neuronal cell bodies in the subtantia nigra pars compacta are poorly understood, aging, genetics, environmental toxicity, and particularly inflammation, represent prominent etiological factors in PD development. Especially, reactive astrocytes, microglial cells, and infiltrating monocyte-derived macrophages play dual beneficial/harmful effects, via a panel of pro- or anti-inflammatory cytokines, chemokines, neurotrophic and neurogenic transcription factors. Notably, with age, microglia may adopt a potent neurotoxic, pro-inflammatory “primed” (M1) phenotype when challenged with inflammatory or neurotoxic stimuli that hamper brain's own restorative potential and inhibit endogenous neurorepair mechanisms. In the last decade we have provided evidence for a major role of microglial crosstalk with astrocytes, mDA neurons and neural stem progenitor cells (NSCs) in the MPTP- (1-methyl-4-phenyl-1,2,3,6-tetrahydropyridine-) mouse model of PD, and identified Wnt/β-catenin signaling, a pivotal morphogen for mDA neurodevelopment, neuroprotection, and neuroinflammatory modulation, as a critical actor in glia-neuron and glia-NSCs crosstalk. With age however, Wnt signaling and glia-NSC-neuron crosstalk become dysfunctional with harmful consequences for mDA neuron plasticity and repair. These findings are of importance given the deregulation of Wnt signaling in PD and the emerging link between most PD related genes, Wnt signaling and inflammation. Especially, in light of the expanding field of microRNAs and inflammatory PD-related genes as modulators of microglial-proinflammatory status, uncovering the complex molecular circuitry linking PD and neuroinflammation will permit the identification of new druggable targets for the cure of the disease. Here we summarize recent findings unveiling major microglial inflammatory and oxidative stress pathways converging in the regulation of Wnt/β-catenin signaling, and reciprocally, the ability of Wnt signaling pathways to modulate microglial activation in PD. Unraveling the key factors and conditons promoting the switch of the proinflammatory M1 microglia status into a neuroprotective and regenerative M2 phenotype will have important consequences for neuroimmune interactions and neuronal outcome under inflammatory and/or neurodegenerative conditions.

## Introduction

Aging is the leading risk factor for the development Parkinson's disease (PD), a most prevalent central nervous system (CNS) movement disorder characterized by the progressive and selective degeneration of midbrain dopaminergic neurons (mDA) of the substantia nigra pars compacta (SNpc) and their terminals in the striatum, the presence of intracellular aggregated inclusions containing α-synuclein (α-Syn), called Lewy bodies (LB), and an abnormal activation of the astroglial cell compartment (Hornykiewicz, 1993; Di Monte and Langston, 1995; Langston et al., 1998, 1999; Table 1).

**Table 1:** Parkinson's disease: from hallmarks to therapy.

**Hallmarks**	**Genetic factors**	**Environmental factors**	**Protective factors**	**Therapy**
**Selective mDA neurodegeneration** in the SNpc with concurrent loss of mDA neuronal afferents to striatum and putamen α**-SYN aggregation** (Lewy bodies and Lewy neurites formation) indicative of ongoing degeneration **Astro and microgliosis** (astrocyte hypertrophy, M1-microglial morphological and functional shift)	**PARK Genes** (α-SYN/PARK1, LRRK2/PARK8§°, PRKN/PARK2§°, VPS35/PARK17§, UCH-L1/PARK5, GBA§ **MAPTau**§° **Dopaminergic related genes (**DA-receptors, DA-transporter, TH, COMT, MAO) **GSK-3**β§° **Xenobiotic Metabolism/Detox-related genes** (P450IID1, CYP1A1, NAT1, HMOX1§°, GST, NQO2) **APOE** **Neurotrophic genes** (NURR1§, NGF, BDNF) **Inflammatory related genes** (iNOS, TNF-α, IL-1β, IL-6, ER-β)§°	**Aging**§° **Rural living** herbicides and pesticides exposure (paraquat, rotenone, organochlorines, carbamates)§°, metal exposure° **Head injuries** **Estrogen deficiency** (women)§° **Infectious diseases** during childhood§° **Maternal factors/ early-life events** (virus, drugs, endotoxins, hormonal deficits)° **Drug-induced parkinsonism** (drug abuse, neuroleptics, calcium-channel blockers)° **miRNAs** (miR-155, miR-7116-5p)°	**Chronic use of NSAIDS** (reduces PD risk)§^*^; ^**^ **Estrogen replacement therapy** (post-menopausal women, OVX animals)^*^ **Dietary factors/life style** (tea, polyphenols, wine components, curcumin, coffee, tobacco)^*^ **Environment (**Exercise, social interactions)§^**^ **miRNA** (miR-7)^*^	**Symptomatic**: L-DOPA or DAergic agents administration (relieve motor symptoms, do not prevent disease progression) **Neuroprotective/symptomatic** (selegiline, rasagiline) **Cell based therapies** (re-introducing DA-producing cells, embryonic, NSCs, treated iPSCs, to replenish DA stores and alleviate/cure PD **Combined therapies (**anti-oxidants, anti-inflammatories, GSK-3β-inhibitors, protective factors to boost endogenous neurogenesis and mDA neuro-restoration)

§ *Wnt/β-CAT dysregulation in the reported conditions*.

° *Activation of microglia and pro-inflammatory mediators in animal models of PD under the reported treatments*.

* *Mitigation/inhibition of microglial activation in animal models of PD under the reported treatments*.

** *Enhanced neurogenesis/synaptic plasticity and glial proliferation*.

The chronic decrease of dopamine storage in the striatum is responsible for the gradual impairment of motor function leading to the classical motor features of PD, which include bradykinesia, rest tremor, rigidity and postural instability. These motor signs are often preceded by nonmotor manifestations such as olfactory dysfunction, autonomic, cognitive and mood function impairments (Langston, 2006).

The causes and mechanisms leading to the progressive and selective mDA neuron death are ill-defined, and so far, there is no cure for PD. Current treatments are centered on dopamine replacement therapy, using the metabolic precursor of dopamine, L-DOPA, or dopamine receptor agonists, albeit they only temporally alleviate the motor symptoms without stopping the ongoing neurodegeneration (Olanow and Schapira, 2013; Obeso et al., 2017, for a comprehensive review). Thus, the ideal therapeutic regimen for PD should combine both symptomatic treatment and neurorestorative interventions aimed at protecting or enhancing the function of DA neurons.

The disease can be divided into sporadic and early-onset familial PD with most (90%) PD cases being sporadic (Ferreira and Massano, 2016) and current evidence indicates that a complex interplay between genetic susceptibility and a panel of environmental factors strongly contribute to PD pathophysiology (Di Monte et al., 2002; Gao and Hong, 2008, 2011; Gao et al., 2011, 2012; Marchetti et al., 2011; Cannon and Greenamyre, 2013; Hirsch et al., 2013; Table 1). Indeed, several genes and many environmental factors impact in the regulation of crucial pathways involved in inflammatory glial activation, mitochondrial function, endoplasmic reticulum stress, autophagic catabolism, protein misfolding and aggregation, that can variously impact in the progressive demise of mDA neurons (Olanow et al., 2003; Abou-Sleiman et al., 2006; Marchetti et al., 2011).

Aging represents the chief risk factor for PD development. With advancing age the function of the nigrostriatal DA system progressively declines leading to neurochemical, morphological and behavioral changes (Boger et al., 2010; Hindle, 2010; de la Fuente-Fernández et al., 2011). Additionally, while nigrostriatal DA neurons are endowed with an extraordinary compensatory/neurorepair capacity, the aging process sharply impair DA neuron plasticity and its ability to recover upon injury (Collier et al., 2007).

Notably, oxidative stress and low-grade inflammation are the hallmarks of aging, and both processes are even further up-regulated upon injury, neurotoxin exposure, male gender and PD genetic mutations (Table 1). With age, microglial cells become “primed,” i.e., capable to produce exacerbated levels of a set of pro-inflammatory mediators when challenged with immune or neurotoxic stimuli. This microglial cell shift to the harmful, M1 phenotype, promotes the release of an array of factors that are detrimental for the vulnerable mDA neurons. Nuclear factor κB (NF-κB), is a key actor and the first signal for inflammasome induction (Codolo et al., 2013), together with major pro-inflammatory cytokines, such as tumor necrosis factor α (TNF-α), interleukin 1β (IL-1β) and IL-6. This inflammatory microenvironment is also associated to oxidative stress mediators such as reactive oxygen (ROS) and nitrogen species (RNS), that in turn amplify microglial activation, which results in increased mDA neuron vulnerability, and/or neuronal death (Olanow et al., 2003; Abou-Sleiman et al., 2006; Hirsch and Hunot, 2009).

Notably, a number of genetic mutations interact with certain risk factors, such as exposure to neurotoxins or endotoxins, then resulting in a further exacerbation of glial activation. In this condition, gene-environment interactions may drive a vicious cycle of oxidative stress and inflammation, contributing to the chronic PD progression (Di Monte et al., 2002; Marchetti and Abbracchio, 2005; Zhang et al., 2005; Whitton, 2007, 2010; Gao and Hong, 2008, 2011; Przedborski, 2010; Tansey and Goldberg, 2010; Gao et al., 2011, 2012; Lastres-Becker et al., 2012; Table 1).

Furthermore, crosstalks between central and peripheral inflammation together with changes in hormonal background with age, may well have further important roles in shaping the final glial response with consequences for neuroprotection/degeneration upon injury (Baba et al., 2005; Marchetti and Abbracchio, 2005; Brochard et al., 2009; Marchetti et al., 2011; Collins et al., 2012; Kannarkat et al., 2013; Chen et al., 2015).

**Figure 1:**
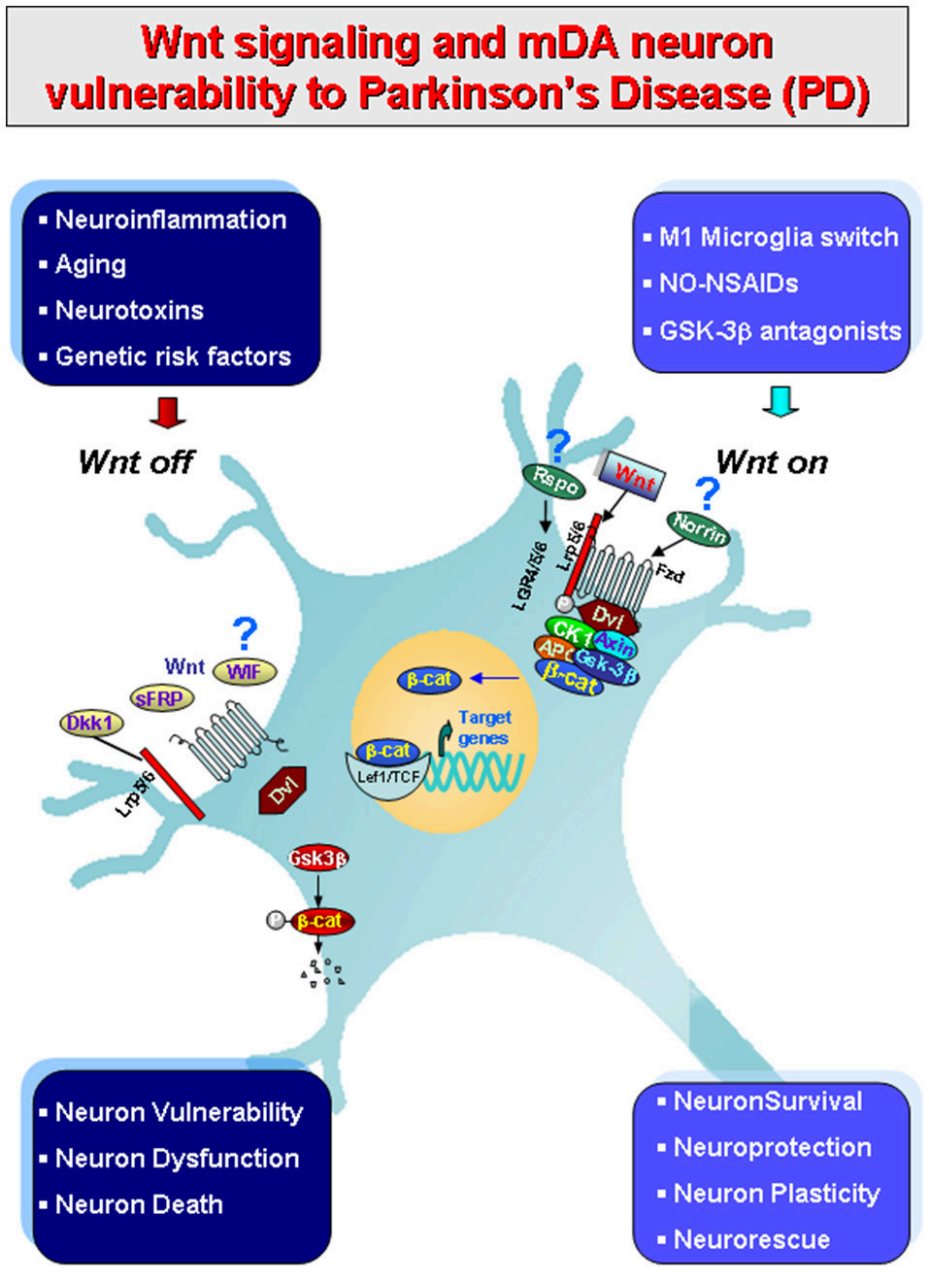
Schematic illustration of gene-environment interactions impacting in mDA neuron survival/protection in the adult midbrain via Wnt1/β-catenin signaling. Major environmental factors including aging, inflammation, neurotoxin exposure including PD neurotoxins (MPTP/MPP+, 6-OHDA), pesticides (rotenone), increased oxidative load as a result of gowth factors (GFs) deprivation in synergy with genetic mutations (see Table 1), may antagonize Wnt/β-catenin signaling (“*Wnt off* ”) in mDA neurons. Up-regulation of active GSK-3β, then lead to β-catenin degradation and increased DA neuron vulnerability/degeneration/apoptosis. By contrast, in the intact midbrain canonical Wnt agonists, such as *Wnt1, Rspo* or *Norrin*, and activation of *Fzd-1* receptors also via exogenous Wnt/β-catenin activation such as GSK-3β antagonist, NO-NSAIDs treatments tors (“*Wnt on*”), contribute to maintain the integrity of mDA neurons via blockade of GSK-3β-induced phosphorylation (P) and proteosomal degradation of the neuronal pool of β-catenin. Stabilized β-catenin can translocate into the nucleus and associate with a family of transcription factors and regulate the expression of Wnt target genes involved in DA neuron survival/plasticity, neuroprotection and repair. β-catenin may also function as a pivotal defense molecule against oxidative stress, and can act as a coactivator for several nuclear receptors involved in the maintenance/protection of DA neurons. The hypothetical contribution of various endogenous Wnt agonists (Respondin, Rspo, Norrin) or antagonists (*Dkkopf, Dkk1, Wif*, frizzled-related proteins, SFRp) are also indicated.

**Figure 2:**
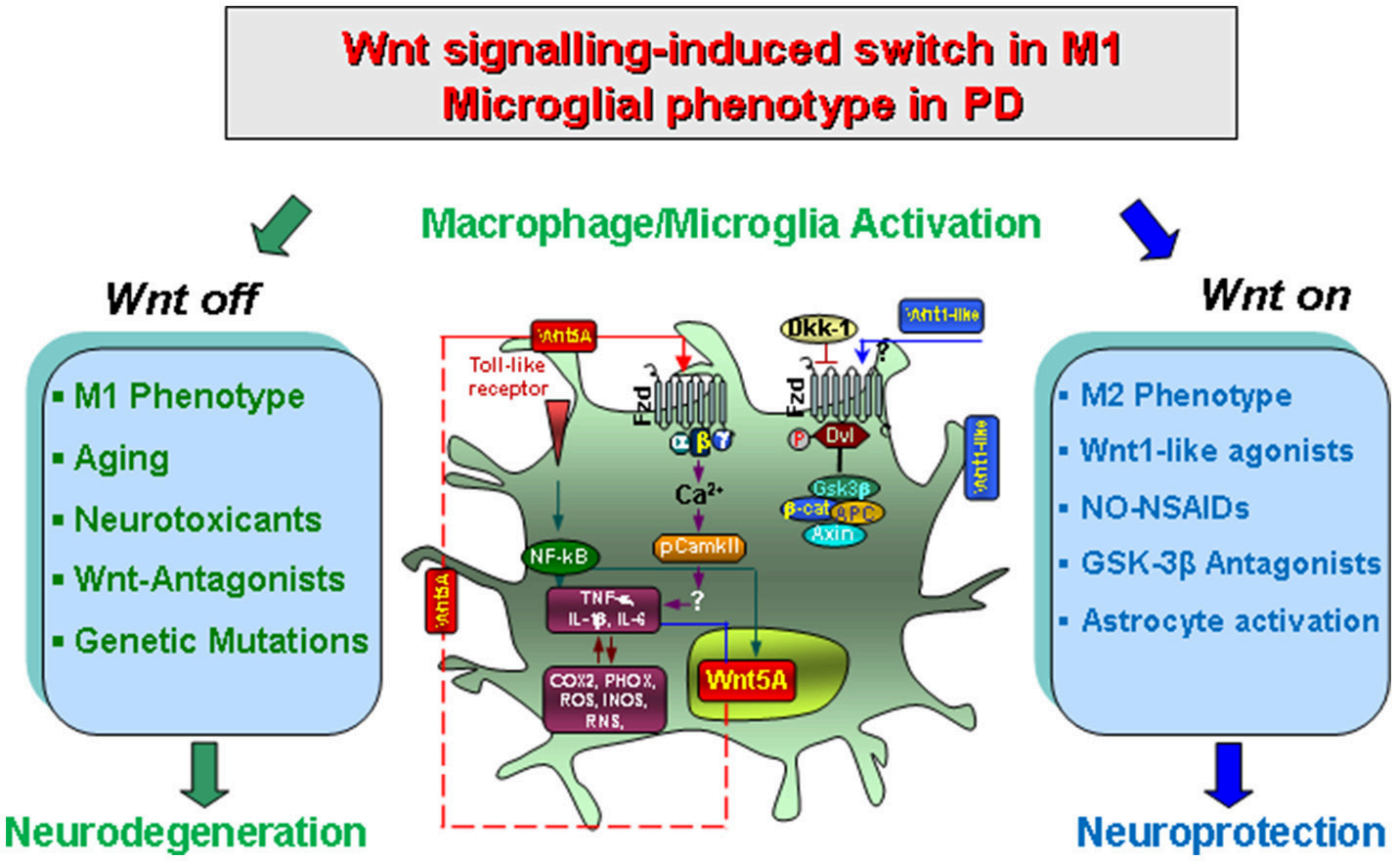
Wnt/β-catenin signaling-induced switch in proinflammatory microglial M1 phenotype in concert with gene-environment interactions in PD. Upon activation by neurotoxins, endotoxins or brain injury and aging, macrophage/microglia produce a panel of pro-inflammatory cytokines (TNF-α and IL-1β) and chemokines (CCL3, CXCl10 and CXCL11), of which Wnt5a constitutes one part of a self-perpetrating cycle, via autocrine Wnt5A/CamKII activation and paracrine stimulation of Th-1- cytokines, iNOS and COX2 (Pereira et al., 2009; Neumann et al., 2010; Halleskog et al., 2012). Up-regulation of microglial PHOX-derived ROS, iNOS-derived NO, and GSK-3β, a known regulator of NF-kB-dependent gene transcription, further exacerbate microglia reaction (Beurel et al., 2010; L'Episcopo et al., 2012, 2013). In addition, astrocyte-derived growth/neurotrophic and anti-oxidant factors including Wnt1, can mitigate the inflammatory milieu and favor a progressive neurorescue program for mDA neurons (Marchetti et al., 2013). However, an exaggerated M1 microglial pro-inflammatory status as observed with age, MPTP exposure, and synergy with different gene and environmental risk factors can impair astrocyte anti-inflammatory and neuroprotective functions also via inhibition of Wnt1 expression and downregulation of anti-oxidant/anti-inflammatory cytoprotective proteins in astrocytes (L'Episcopo et al., 2013). Modified from Marchetti and Pluchino (2013), with permission.

We recently provided evidence that the Wnt/β-catenin signaling pathway, a chief player in neurodevelopmental processes (Ciani and Salinas, 2005; Clevers, 2006; Prakash and Wurst, 2006; Salinas, 2012; Joksimovic and Awatramani, 2014; Wurst and Prakash, 2014; Zhang et al., 2015), is crucially involved in the physiopathology of nigrostriatal DA neurons (L'Episcopo et al., 2011a,b; Marchetti et al., 2013; Harvey and Marchetti, 2014). Furthermore, growing evidence indicates the contribution of Wnt signaling in the modulation of inflammation via bidirectional glia-neuron crosstalk in PD (L'Episcopo et al., 2011a,b, 2014a,b; Marchetti and Pluchino, 2013; Marchetti et al., 2013; Figure 1). Then, astrocytes and macrophage/microglial cells in the brain, and immune cells in the periphery express Wnts and harbor a panel of Wnt's receptors thereby modulating in an autocrine/paracrine fashion immune responses both at central and peripheral levels (Staal et al., 2008; Pereira et al., 2009; Neumann et al., 2010; Halleskog et al., 2011, 2012; Kilander et al., 2011; L'Episcopo et al., 2011a, 2012, 2013, 2014a; Halleskog and Schulte, 2013a,b; Marchetti and Pluchino, 2013). In turn, Wnt receptors are present in mDA neurons and Wnt/β-catenin signaling activation exert robust neuroprotective effects (L'Episcopo et al., 2011a,b, 2012, 2013, 2014a,b; Harvey and Marchetti, 2014; Figures 1, 2).

**Figure 3:**
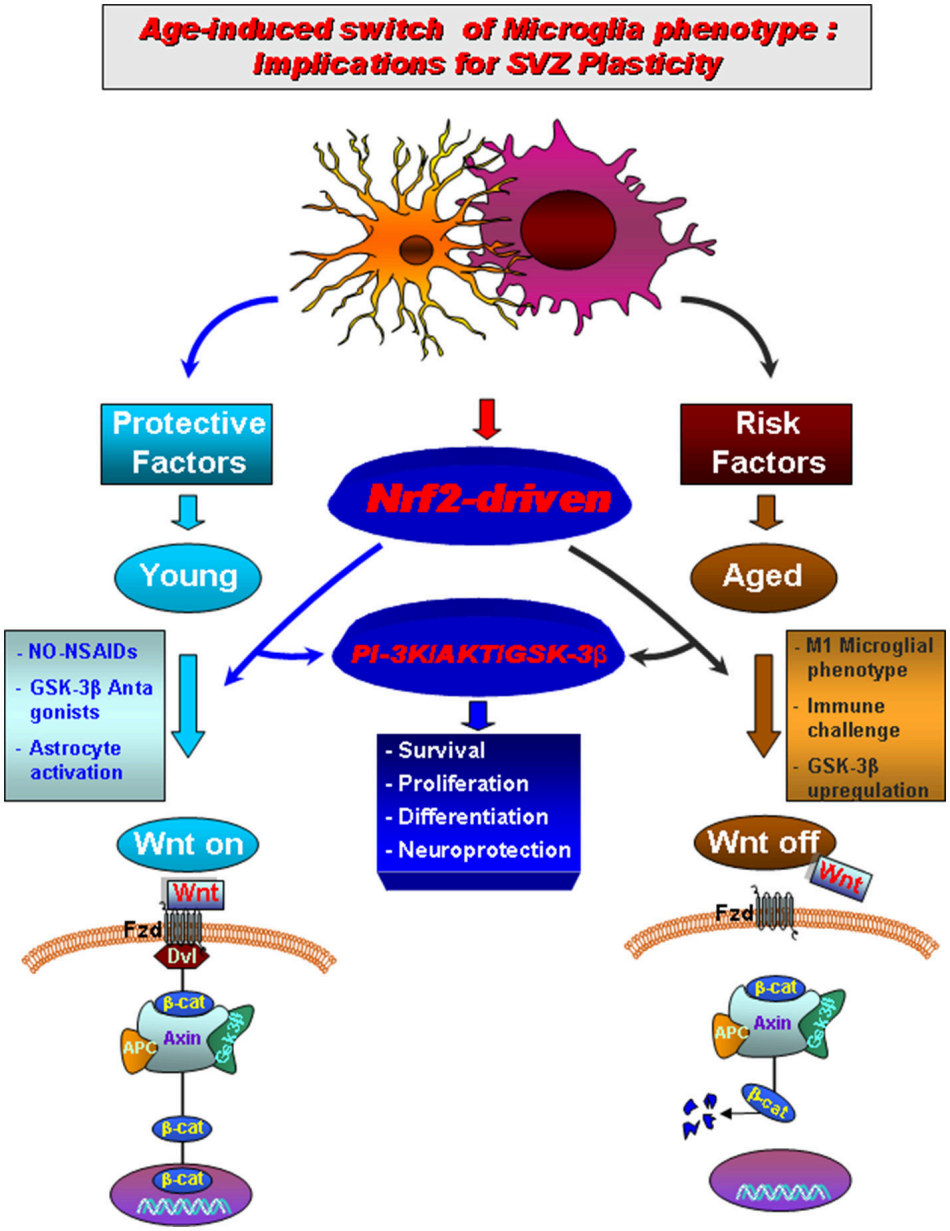
Aging-induced M1 proinflammatory phenotype promotes *Nrf2-ARE* pathway disruption in the subventricular zone (SVZ) driving neurogenic impairment in parkinsonian mice via *PI3K-Wnt/*β*-catenin* dysregulation. In young mice a regulatory circuit linking microglial activation and pro-inflammatory cytokine to *Nrf2-ARE* protective pathway in SVZ, provides an efficient self-adaptive mechanism against inflammatory/neurotoxin-induced oxidative stress. In addition to govern the redox balance within the SVZ niche, *Nrf2*-induced *Hmox* target gene may simultaneously protect astrocytes, thereby up-regulating the expression of vital *Wnt* signaling elements switching-on key components required for maintaining SVZ cells in a proliferative state, promote differentiation and/or for exerting neuroprotective effects. Crosstalk between two pivotal pathways, the *PI3-K/Akt/GSK-3*β and *Wnt/*β*-catenin* signaling cascades appear to cooperate to finely control the transcriptional activator, β*-catenin*, in turn representing a point of convergence to direct proliferation/differentiation/survival in SVZ stem niche. Importantly, SVZ “rejuvenation” may have beneficial consequences for DAergic neuroprotection, and viceversa. Astrocytes (blue), neuroblasts (red), transit-aplifying cells (yellow) and ependymal (purple) cells in SVZ niche are schematically illustrated (modified from L'Episcopo et al., 2013, with permission).

Microglia and astrocyte-microglia crosstalk also modulate the brain'own regenerative/neurorestorative potential, regulating adult neural stem/progenitor cell (NSC) plasticity in neurogenic niches (Pluchino et al., 2005; Jakubs et al., 2008; Ekdahl et al., 2009; Schwartz et al., 2009; Ehninger et al., 2011; Ekdhal, 2012; Kokaia et al., 2012; L'Episcopo et al., 2012, 2013; Marchetti and Pluchino, 2013). However, aging, inflammation and PD, exacerbating microglia M1 phenotype impair NSCs proliferation and neuronal differentiation and inhibit Wnt/β-catenin signaling (Freundlieb et al., 2006; Okamoto et al., 2011; L'Episcopo et al., 2012; 2013), with harmful consequences for mDA neuron recovery and repair upon injury (L'Episcopo et al., 2013, 2014a,b; Figure 3).

Recently, in several neurodegenerative diseases, including PD, a dysregulation of non-coding RNAs (ncRNAs) levels has been reported (Sonntag, 2010). MicroRNAs (miRNAs) are the most studied class of ncRNAs, which play key roles in normal cellular physiology as well as in pathogenesis, including PD pathogenesis (Bartel, 2004; Soifer et al., 2007; Bian and Sun, 2011). Of special importance for the present work, different miRNAs are increasingly being appreciated for their ability to modulate the microglial inflammatory response in PD, with novel potential therapeutic implications for regulating the inflammatory response during PD progression (Thome et al., 2016).

In this work we will first introduce the role of neuroinflammation in PD, with a specific focus on microglia-astrocyte-neuron crosstalk. Particularly, the role of gene-environment interactions such as aging, neurotoxins and inflammogen exposure and their influence in microglial polarization and Wnt signaling together with the interplay between mRNAs and miRNA modulatory effects will be next addressed.

## Neuroinflammation and PD: the key role of microglial-astrocyte-neuron tripartite crosstalk

A body of evidence from epidemiological and post-mortem studies in human PD brains, coupled to accumulating data in experimental models of PD in either non-human primates and rodent PD models, clearly indicates that neuroinflammatory glial-mediated mechanisms are chiefly involved in PD pathophysiology, playing a dual benefical/harmful role (Marchetti and Abbracchio, 2005; Marchetti et al., 2005a,b,c; McGeer and McGeer, 2008).

Although the “primum movens” initiating the inflammatory response and the causal relationship between the two phenomena remain to be fully clarified, it is recognized that neuronal degeneration itself, particularly aggregated α-Syn (a core feature of both sporadic and familial forms of PD) (Bendor et al., 2013), released early in the disease process by the injured DA neurons, may act as an endogenous disease-related signal, activating glial cells to release a variety of pro-inflammatory molecules, promoting microglia exacerbation and neuronal cell death (Zhang et al., 2005; Whitton, 2007; Gao et al., 2011; Codolo et al., 2013; Sanchez-Guajardo et al., 2013). In turn, neuroinflammatory glial activation has been suggested also to contribute via the promotion of a prion-like behavior of misfolded α-Syn propagation (Lema Tomé et al., 2012).

The possibility that an early dysregulated microglial pro-inflammatory phenotype contributes to progressive nigrostriatal degeneration in PD has received increasing attention in the light of the implications for preventive and therapeutic strategies for PD (Marchetti and Abbracchio, 2005; Zhang et al., 2005; Whitton, 2007, 2010; Gao and Hong, 2008; Koprich et al., 2008; Hirsch and Hunot, 2009; Deleidi et al., 2010; L'Episcopo et al., 2010a,b, 2011c). Hence, positron emission tomography imaging studies employing microglia-specific markers support an early involvement and cerebral propagation of neuroinflammation in PD (Gerhard et al., 2006; Ouchi et al., 2009; Pradhan and Andreasson, 2013). Prospective studies suggest that inflammatory processes can modulate PD risk in humans, as higher plasma concentrations of the pro-inflammatory cytokine interleukin-6 (IL-6) increased the risk of developing PD whereas chronic nonsteroidal anti-inflammatory drug (NSAID) regimens reduced the incidence of PD by 46% (Chen et al., 2003, 2005; Schiess, 2003). Importantly, the association of late-onset sporadic PD with certain genetic variants in the region of chromosome 6 that specifies the human leukocyte antigens (HLAs), which are crucial for immune function in humans (Hamza et al., 2010), have been further strengthened using Genome-wide association studies (GWAS) (Latourelle et al., 2012). Especially, meta-analyses by the International Parkinson Disease Genomics Consortium et al. (2011), supported the evidence for association of five previously reported risk loci near the genes for alpha-synuclein (*SNCA*), microtubule associated protein tau (*MAPT*), cyclin G-associated kinase (*GAK*), beta-glucocerebrosidase (*GBA*), and *HLA* locus (HLA).

Consistent with the inflammation hypothesis, experimental evidences in different PD rodent models indicate significant neuroprotective effects exerted by different immunomodulatory drugs including non steroidal anti-inflammatory drugs (NSAIDs). However, there are some conflicting results in the ability of the different NSAIDs to effectively protect mDA neurons against neurotoxic insults, likely due to the dual (beneficial/harmful) effects of inflammation, the timing of the NSAID treatment (i.e., before or after mDA neuron injury), and the specific properties of the different NSAIDs (reviewed by Marchetti and Abbracchio, 2005; Fiorucci and Antonelli, 2006; Esposito et al., 2007; Whitton, 2007, 2010; L'Episcopo et al., 2010a,b, 2011c; Pradhan and Andreasson, 2013).

Within this scenario the major players are the microglial cells, the reactive astrocytes, and the infiltrating monocyte-derived macrophages (Depboylu et al., 2012). Notably, microglia are highly pleiotropic cells and dynamically shift between a quiescent (termed M2)-to moderate or highly activated (termed M1) states, depending on the triggering mechanisms and the duration of the insult (Kreutzberg, 1996; Streit, 2002; Perry and Teeling, 2013). In the basal M2 state, microglia have anti-inflammatory and neuron-reparative roles, protecting neighboring cells by removing cell debris and releasing trophic factors for brain repair. Upon injury or immune challenges, activated M1 microglia proliferate and participate in clearing cell debris at early stages, but may exacerbate brain injury by producing neurotoxic substances, especially when overactivated for prolonged times (Perry and Teeling, 2013). In these conditions, microglia release a variety of pro-inflammatory mediators that can become detrimental to neuronal survival. Major players are the transcription factor NF-κB and activator protein-1 (AP-1) chiefly involved in the induction of multiple inflammatory genes involved in the inflammatory response. Particularly, among glial cytotoxic molecules, inducible NO synthase (iNOS)-derived NO, superoxide from the plasma membrane NADPH oxidase, cyclooxygenase 2 (COX2)-derived prostaglandin E2, associated with a number of potent inflammatory cytokines, including TNF-α, IL-1β, IL-6, and IFN-γ shown to exert detrimental effects in mDA neurons (Sriram et al., 2002, 2006; Teismann et al., 2003; Whitton, 2007, 2010; Gao and Hong, 2008; Hirsch and Hunot, 2009).

As astrocytes are concerned, they are prominent players both in health and disease (Sofroniew and Vinters, 2010). They contribute to a panel of key functions in the CNS, including the provision of trophic support to neurons, clearance of debris, as well as the modulation of synapse formation and function, energy metabolism, and in particular the defense against oxidative stress (see Bélanger and Magistretti, 2009). For example, efflux of GSH from astrocytes mediated by the ATP-dependent transporter, multidrug-resistance associated protein (Mrp1) is involved in the dynamic response to the changing redox mileu (Gennuso et al., 2004). The expression and activation of anti-oxidant response element (ARE) represent a key feature of astrocyte neuroprotective effects. Oxidative stress can up-regulate enhance expression and binding of astrocytic NF-E2-related factor 2 (*Nrf2*), which translocates to the nucleus and binds to ARE. Importantly, binding to ARE up-regulates a cluster of anti-oxidant genes, including those for GSH, as well as anti-oxidant, anti-inflammatory and cytoprotective genes, such as *Heme oxygenase1* (*Hmox*) (Chen et al., 2009).

Astrocytes' modulation of the local microenvironment is complemented by the expression and release of a variety of growth and neurotrophic factors and a number of pro/anti-inflammatory mediators and anti-oxidant molecules (see Marchetti et al., 2013). Furthermore, astrocytes can contribute to cell genesis both as stem cells and as important cellular elements of the neurogenic microenvironment, with implications for self-recovery/neurorepair (Alvarez-Buylla et al., 2001). Upon injury, astrocytes can transform into “reactive” astrocytes (Ras), that can fulfill both neuroprotective or neurotoxic functions. Ras are characterized by up-regulation of several molecules including GFAP and S100, they express receptors involved in innate immunity (e.g., Toll-like receptors), participating in the regulation of astrocyte response to injury. In addition, Ras express receptors for growth factors, chemokines, hormones, and produce a wide array of chemokines and cytokines that act as immune mediators in cooperation with those produced by microglia (Marchetti et al., 2005a,b).

Thanks to the shared receptors for neurotransmitters, hormones, neuromodulators, neuropeptides and immune regulatory molecules, neurons, astrocyte and microglial cells can talk with each other and sense the changing microenvironment. Then, glial-neuron crosstalk is essential for maintaining CNS homeostasis during physiological, and particularly under neurodegenerative and inflammatory conditions. Especially, astrocyte-microglia crosstalk plays a pivotal role, aimed at reducing or inhibiting any exacerbated inflammatory/oxidative response in the brain (Bélanger and Magistretti, 2009; Marchetti and Pluchino, 2013). This appears of particular importance given that microglial cells in the SNpc are more abundant (about 4.5-fold) as compared to any other brain region, while SNpc-DA neurons have reduced anti-oxidant potential, and the redox chemistry of dopamine present in the cytoplasm could be enhanced by an exacerbation of ROS production, leading to the formation of toxic dopamine metabolites. All together these conditions predispose mDA neurons to vulnerability to inflammatory/oxidative attacks (Abou-Sleiman et al., 2006; Whitton, 2007, 2010; McGeer and McGeer, 2008; Tansey and Goldberg, 2010; Taylor et al., 2013).

Consequently, the microglial M1 proinflammatory status is tightly linked to astrocyte-microglia and neuron-glia interactions through a number of mechanisms and a panel of inhibitory receptors that restrain microglial activation. For example, CD200, a transmembrane glycoprotein expressed on neurons, can survey glial activation status via its binding to CD200R (Wang et al., 2011; Zhang S. et al., 2011). When CD200-CD200R engagement is disrupted, this can lead to an abnormal activation of microglia and consequent pathological changes. Importantly, microglia harbor hormonal receptors (i.e., for glucocorticoid hormones, GRs, and for estrogens, ERs) contributing to limit microglial overactivation via the blockade of principal inflammatory pathways, particularly NFκB signaling and the iNOS-NO pathway generating elevated concentrations of proinflammatory cytokines and RNS (Marchetti et al., 2002, 2005a,b, 2011; Vegeto et al., 2003; Morale et al., 2004, 2006, 2008; L'Episcopo et al., 2010a).

Upon exposure to the PD neurotoxins including 6-OHDA- or MPTP, glia-neuron and astrocyte-microglia crosstalk play decisive roles in dicating the severity of the nigrostriatal lesion and the repair capacity of the dysfunctional mDA neurons, according to the SNpc microenviroment, the age and sex of the host. In humans and non-human primates exposed to MPTP, the presence of Ras in the SN lasts for 1–16 years following the initial insult, but the biological significance of Ras is not completely understood (Collier et al., 2007; Barcia et al., 2013). Of note, however, in the presence of chronic microglia over-activation, Ras can loose both neuroprotective and neurorepair properties with harmfull consequences for the dysfunctional mDA neurons. Hence, a prolonged dysfunction of astrocytes and activation of microglia can accelerate the degeneration of SNpc DA neurons, blocking the compensatory mechanisms of mDA neuron repair during early dysfunction induced by 6-OHDA lesion in rats, thus underlying the important role of astrocytes in early degeneration of mDA neurons (Kuter et al., 2017). Importantly enough, in analogy to the M1/M2 macrophage nomenclature, neuroinflammation and brain injury were shown to promote two different types of Ras termed A1 and A2, with the A1-Ras phenotype promoting destructive effects, and the A2 state exerting neuroprotective roles (Liddelow et al., 2017). Then, when stimulated by LPS, activated M1 microglia secreting proinflammatory cytokines, such as IL-1β and TNF- α, contribute to the promotion of the Ras A1 phenotype leading to the inhibition of astrocyte's ability to promote neuronal survival, outgrowth, synaptogenesis and phagocytosis, and to induce the death of neurons and oligodendrocytes (Liddelow et al., 2017).

As far as the cytotoxic mechanism(s) involved in mDA neuron death, of specific mention, when iNOS and NADPH oxidase are present together, then a potent toxin, peroxynitrite (ONOO-), is produced which promotes the nitration of proteins, like tyrosine, with further production of hydroxyl radicals. For example, the production of the free radical NO together peroxynitrite are sought to be involved in mDA neuron demise (Gao and Hong, 2008; Hirsch and Hunot, 2009; Taylor et al., 2013). Regarding the cytokines, TNF-α can directly activate TNF receptors (TNF-Rs) present on mDA neurons and trigger a pro-apoptotic cell death pathway. Also, the TNF-α-dependent proinflammatory microenvironment within the SN is further amplified by increased oxidative stress through activation of PHOX, the expression of COX-2 and the stimulation of iNOS. The resulting production of ROS, RNS, excitotoxic mediators, such as glutamate and a panel of reactive molecules, further amplify the inflammatory reaction engendering a vicious cycle, resulting in the exacerbation of the neurodegenerative process (Whitton, 2007; More et al., 2013).

Importantly enough, both microglia and astrocytes are dysfunctional with advancing age. Hence both cell types show region-specific changes in morphology such as structural deterioration or dystrophy, decreased expression of growth/neurotrophic factors and an impaired phagocytic activity in face of increased marker expression and up-regulation of pro-inflammatory molecules, all of which are associated to a gradual loss of astrocyte and microglia neuroprotective capacity (Mouton et al., 2002; Streit et al., 2004; Morale et al., 2006; Damani et al., 2010; L'Episcopo et al., 2011c; Njie et al., 2012). Reportedly, microglial switch to a so-called “primed” status, endowed with a strong neurotoxic, pro-inflammatory M1 phenotype (Streit, 2010; Njie et al., 2012) with cytotoxic influences for mDA neuron health (L'Episcopo et al., 2011c).

Especially, work from our laboratory indicates that age-dependent changes in the glial compartment results in a dysregulation of glia-neuron crosstalk and play key roles in the impairment of nigrostriatal DA plasticity (L'Episcopo et al., 2011a,b,c). In fact, increased vulnerability and mDA neuron death are observed after exposure of aging mice to neurotoxin or inflammatory triggers, supporting that glia dysfunction with age represents a primary risk factor and a common final pathway for neurodegenerative disorders in general and for PD in particular (L'Episcopo et al., 2010a,b). Notably, mDA neuron numbers and striatal innervation as well as DA release and motor deficits show a remarkable ability to recover after acute or chronic administration of MPTP or 6-OHDA in young rodents and non-human primates, but this adaptive capacity is lost with age (Collier et al., 2007; Boger et al., 2010; Hindle, 2010; de la Fuente-Fernández et al., 2011; L'Episcopo et al., 2011a,b; Blandini and Armentero, 2012; Bové and Perier, 2012).

In addition to glial cells, other cells may also participate in the neuroinflammatory processes in PD, as increasing evidence demonstrates the involvement of both innate and adaptive immune responses in the pathophysiology of PD (Baba et al., 2005; Orr et al., 2005; Brochard et al., 2009; Collins et al., 2012; Kannarkat et al., 2013; Chen et al., 2015). The infiltration of CD4/CD8 T-cells has been reported both in the SN of PD patients and in animal models of PD, together with alterations in the peripheral T-cell pool is altered in PD, with potential interactions with the local SN microglial environment promoting further exacerbation of M1 phenotype (Brochard et al., 2009; Barcia et al., 2013; reviewed by Sanchez-Guajardo et al., 2013). Cytokine and chemokine expression are also upregulated in peripheral blood mononuclear cells (PBMCs) in PD patients.

Of specific mention, with age, there is an up-regulation of several inflammatory markers in the periphery associated to a dysfunctional blood-brain barrier (BBB), resulting in increased crosstalk between the CNS and peripheral immune system (Cunningham et al., 2005). This increased sytemic proinflammatory status may then trigger inflammatory glial responses, associated to an exaggerated production of various inflammatory molecules such as TNF-α, IL-1β, coupled to production of high levels of ROS and RNS, promoting a vicious cycle of oxidative stress and inflammation leading to neuronal death (Streit et al., 2004; Cunningham et al., 2005; Flanary, 2005; Godbout et al., 2005; Flanary et al., 2007; Hu et al., 2008; Pott Godoy et al., 2008; Henry et al., 2009; Damani et al., 2010; L'Episcopo et al., 2010a,b, 2011c; Streit, 2010; Njie et al., 2012). Hence, young adult and aging mice respond in a strikingly different way when an acute subthreshold dose of LPS was systemically administrated, as a single LPS injection in old mice resulted in exacerbated production of pro-inflammatory markers both at central and peripheral levels. This general proinflammatory status then triggered a slow but progressive mDA neuron loss during the entire lifespan of the mice (L'Episcopo et al., 2011c). By contrast, the concomitant treatment with the NO-releasing NSAID, Flurbiprofen (NO-Flurbi) (Fiorucci and Antonelli, 2006) was capable to mitigate the exacerbated M1 microglia pro-inflammatory phenotype induced by the systemic neurotoxic challenge, resulting in a lifelong protection of SNpc DA neurons (L'Episcopo et al., 2011c; Figure 2).

All together these findings support the contention that glia-neuron crosstalk in the brain, complemented by a proinflammatory status at peripheral levels, may represent a major risk factor and final common pathway for mDA neuron vulnerability to PD degeneration. Additionally, they provide a mechanistic link between microglial M1 pro-inflammatory status of aging mice, microglia-DA neuron crosstalk and DA cell demise, and offer a therapeutical window of opportunity to rescue mDA neurons from inflammation-mediated neurodegeneration of old mice by targeting the microglial pro-inflammatory phenotype (Figures 1, 2). Within this frame, the role of astrocytes clearly appear decisive, since they can either cooperate with microglia to exacerbate M1 phenotype and the consequent neurotoxicity, or in the contrary, they can downregulate microglia activation, to support the imperiled/dysfunctional mDA neurons and activate intrinsic cues for DA neuroprepair/neurorestoration (Marchetti et al., 2013; L'Episcopo et al., 2014a,b).

## The Wnt/β-catenin signaling pathway: a “new entry” in glia-neuron dialogue

Emerging evidence of the last decades points to *Wingless-type MMTV integration site* (*Wnt*) signaling, a highly conserved pathway across species, as a crucial regulator of a multitude of CNS functions both during development and in the adult brain. Here we will first introduce briefly Wnt signaling and the pathways operating at the mDA neurons, astrocytes and microglial levels.

Wnts are secreted lipid-modified glycoproteins that regulate stem cell self-renewal, differentiation, and cell-to-cell communication during embryonic development and in adult tissues. The activation of Wnt signaling is a complex and well regulated process that relies on the expression of a specific Wnt ligand, the concomitant presence of endogenous/exogenous Wnt signaling regulators, the expression of a particular subtype of Frizzled (Fzd) family receptors, the coreceptors, and the specific cellular context (Gordon and Nusse, 2006; Angers and Moon, 2009; Salinas, 2012; van Amerongen, 2012; Willert and Nusse, 2012; and Wnt homepage: http://www.stanford.edu/~rnusse/wntwindow.html). There are 19 mammalian Wnt genes and 15 receptors and co-receptors distributed over seven protein families in mammals (Niehrs, 2012). Wnt proteins are recognized to activate two major branches of Wnt signaling pathways, the so called “canonical” Wnt/β-catenin (activated by the *Wnt1* class of ligands, Wnt2, Wnt3, Wnt3a, Wnt8, and Wnt8a) and the “non-canonical” that includes the Wnt/PCP and Wnt/Ca^2+^ pathways (activated by Wnt5a class, that includes Wnt4, Wnt5a, Wnt5b, Wnt6, Wnt7a, and Wnt11) (Willert and Nusse, 2012). However, such description appears an oversimplification, since in some instances a same Wnt ligand can activate different pathways depending on the presence of the receptors and coreceptors, the endogenous activators or inhibitors, as well as the specific cellular context. While a detailed discussion of Wnt signaling components is beyond the scope of this work (see Marchetti and Pluchino, 2013), we will summarize the principal actors of Wnt/β-catenin pathway, the most well-characterized Wnt pathway that plays a vital role in mDA neurodevelopment, mDA neuroprotection and regeneration (Harvey and Marchetti, 2014; Figure 1).

Notably, in the canonical Wnt pathway, β-catenin and GSK3β (glycogen synthase kinase 3β) are the key players (Clevers and Nusse, 2012). When specific Wnt1-like ligands are absent (i.e., in the “*Wnt off”* state), the concentration of cytoplasmic β-catenin is maintained at low levels via the constant targeting by a multiprotein destruction complex, composed of two scaffold proteins, Axin and APC (adenomatous polyposis coli), which support the phosphorylation of β-catenin by CK1α (casein kinase 1 α) and GSK3β. As a next step, the phosphorylation of β-catenin results in its recognition and ubiquitination by the E3 ubiquitin ligase β-TrCP (β-transducin repeats containing protein), leading to β-catenin proteasomal degradation. Under such conditions, the nuclear transcription factor lymphoid enhancer-binding factor/T cell-specific (LEF/TCF) is associated with Groucho and represses target gene expression (Roose et al., 1998). In the “*Wnt off* ” state, the phosphorylation of β-catenin in mDA neurons negatively impact both in the survival and protection against a variety of noxious insults (L'Episcopo et al., 2011a,b; Figure 1, and next section).

By contrast, binding of Wnt1-like ligands to Fzd receptor and its co-receptor to the low-density LRP (lipoprotein receptor-related protein)5/6 (i.e., in the “*Wnt on”* state), this results in the formation of large multiprotein aggregates (Bilic et al., 2007), called signalosomes, that are involved in the prevention of β-catenin proteasomal degradation (Zeng et al., 2008). Hence, the kinase activity of GSK3-β is inhibited, leading to the stabilization of cytosolic β-catenin, which then accumulates and translocates to the nucleus to regulate transcription via transcription factor then the TCF/LEF family (Clevers, 2006). Nuclear β-catenin then displaces Groucho and forms a complex with tissue-specific transcriptional activators, and converts LEF/TCF from a transcriptional repressor to an activator that turns on Wnt-dependent gene expression in a very cell-type-specific manner (Mosimann et al., 2009; Cadigan and Waterman, 2012). In mDA neurons, nuclear β-catenin activates Wnt1-dependent genes involved in mDA neuron specification, survival and protection (L'Episcopo et al., 2011a,b, 2012, 2013, 2014a,b; Wei et al., 2012; Figure 1).

Notably, GSK-3β is a serine/threonine protein kinase, that besides its central role in the Wnt/β-catenin pathway, is recognized to play key roles in a variety of cellular processes via a panel of signaling pathways that are crucial for inflammation and oxidative stress, cell proliferation, stem cell renewal and apoptosis/neuronal survival, amongst others (Grimes and Jope, 2001; Jope et al., 2007; Kim et al., 2009; Beurel et al., 2010; Phukan et al., 2010; Beurel, 2011; Kim and Snider, 2011; King et al., 2013). Especially, in the “Wnt off” state, activation of GSK-3β in mDA neurons represents a critical step in SNpc neuron demise upon MPTP-induced neuronal cell death both *in vitro* and *in vivo* (Chen et al., 2004; Duka et al., 2009; Petit-Paitel et al., 2009; L'Episcopo et al., 2011a,b). Additionally, in glial cells, Wn/β-catenin antagonism results in GSK-3β activation and exacerbation of glia activation associated to the production of proinflammatory mediators with consequent glial-dependent neurotoxicity (L'Episcopo et al., 2016 and discussed in next sections).

The β-catenin independent, so called “*non canonical*” Wnts ligands, signal through Fzd receptors as well as members of the receptor tyrosine kinase-like orphan receptor (Ror) family and the Wnt modifier, receptor-like tyrosine kinase (Ryk). This pathway leads to changes in cell polarity and migration and is mediated by Ca^2+^influx as well as activation of the small GTPases, RhoA, Cdc42 and Rac (van Amerongen et al., 2008; Angers and Moon, 2009; van Amerongen, 2012). However, such classifications are not rigid since these pathways can overlap or influence/crosstalk or antagonize β-catenin-dependent signaling, thereby constituting a further regulatory step in the control of Wnt signaling (Angers and Moon, 2009; Glinka et al., 2011).

Remarkably, approximately 400 genes involved in cell growth, differentiation, apoptosis, survival and immune functions are regulated by the Wnt/β-catenin signaling, and in view of its multifunctional roles, this pathway is counter-modulated by different endogenous regulators which include the *Dickkopf* (*Dkk)* family (*Dkk-1*, -2, -3, and -4 and soggy), and secreted frizzled-related proteins (Sfrps) considered as both negative and positive Wnt signaling regulators (Bovolenta et al., 2008; van Amerongen et al., 2008; Angers and Moon, 2009).

All together, potential interactions between Wnt ligands, their receptors and downstream effectors, coupled to crosstalks between the *canonical* and *non-canonical* branches of Wnt signaling anticipates the level complexity of the Wnt signaling machinery. Furthermore, given the in involvement of Wnt signaling in a multitude of developmental processes and the maintenance of adult tissue homeostasis, not surprisingly, an aberrant regulation of this pathway has been linked with a variety of diseases, including cancer, inflammatory, metabolic, or neurodegenerative diseases (Clevers and Nusse, 2012).

## The Wnt1/β-catenin-inflammatory connection for mDA neuroprotection, neurorepair and neurorestoration

Wnt1 is a unique critical morphogen for mDA neurodevelopment and activation of the Wnt1/β-catenin signaling is required for mDA neuron specification (Arenas, 2014; Joksimovic and Awatramani, 2014; Wurst and Prakash, 2014; Toledo et al., 2017). This chief role of Wnt1 is maintained throught life in the adult midbrain, where Wnt1 contributes to the maintenance of SNpc DA neuron survival, neuronal function and synaptic integrity, in promoting the activation of Nurr1^+^ post-mitotic mDA neuroprecursors, in favoring neuroprotection and neurorestoration in the injured PD midbrain and up-regulating adult neurogenesis in neurogenic niches, via glia-neuron and glia-NSCs crosstalk (Inestrosa and Arenas, 2010; L'Episcopo et al., 2011a,b, 2012, 2013, 2014a; Galli et al., 2014; Harvey and Marchetti, 2014; Zhang et al., 2015).

Indeed, astroglial cells are a major source of Wnts and harbor a panel Fzd receptors, that play roles in bidirectional astrocyte-neuron and astrocyte-microglia crosstalk. Work from our laboratory obtained *in vivo* in rodent models of PD, as well as *in vitro*, in primary mesencephalic neuron-astrocyte and astrocyte-microglia coculture systems, indicates that during MPTP injury, in the inflammed midbrain, microglial-derived chemokines induce Wnt1 in astrocytes, and this Wnt1 up-regulation activates canonical Wnt/β-catenin signaling in mDA neurons (L'Episcopo et al., 2011a; Figure 1), as an intrinsic neurorescue response, in turn responsible for mDA neuroprotection against a variety of insults, such as oxidative stress and PD neuroxins (i.e., MPP+ or 6-OHDA) (L'Episcopo et al., 2011a,b; Marchetti et al., 2013). Astrocyte-derived Wnt1 ability to promote neuroprotection is mimicked by specific GSK-3β antagonists and efficiently counteracted by down-regulating Wnt1 expression in astrocytes or inhibiting Wnt/β-catenin pathway activation in mDA neurons with either molecular (short hairpin RNA silencing Wnt1 in astrocytes, Fzd1-knock down with antisense-RNAs, β-catenin silencing in mDA neurons) or pharmacological approaches (inhibiton of Wnt/β-catenin signaling with Dkk1, Sfrps, or Wnt1-Abs, L'Episcopo et al., 2011a,b; Marchetti et al., 2013).

However with the aging process, Wnt signaling declines, leading to dysfunctional neuron-astrocyte and astrocyte-microglia crosstalk (L'Episcopo et al., 2011a,b,c, 2013, 2014a; Okamoto et al., 2011; Marchetti et al., 2013; Seib et al., 2013). Hence, the aged microglia proinflammatory status coupled to the exposure to PD neurotoxins markedly inhibit Wnt1 expression in midbrain astrocytes, with a concomitant downregulation of β-catenin and Fzd-1 receptors in mDA neurons, thereby counteracting both the neurotrophic and proneurogenic potential of astrocytes (L'Episcopo et al., 2011a,b,c, 2012, 2013, 2014a,b).

Of special importance, activation of Wnt signaling also impact in glia functionality, given that Wnt signaling may both promote or down-modulate macrophage/microglial activation and the production of proinflammatory mediators. For example, the “*canonical”* Wnt-3a ligand, and the “*non canonical”* Wnt-5a, can both induce a pro-inflammatory response in primary mouse microglia, *in vitro* (Halleskog et al., 2012; Halleskog and Schulte, 2013a). On the other hand, after LPS- induced proinflammatory transformation of microglia, both Wnt-3a and Wnt-5a exerted a dose-dependent decrease in the pro-inflammatory marker, COX2 (Halleskog and Schulte, 2013a), thereby suggesting that the inflammatory microenvironment plays an important role in dictating the outcome of microglial response to Wnts (Marchetti and Pluchino, 2013).

Likewise, in peripheral macrophages, both Wnt-3a and Wnt-5a can drive a pro-inflammatory transformation with increased production of pro-inflammatory cytokines, such as TNF-α (Pereira et al., 2009). Of special interest, however, in *mycobacterium-* infected macrophages, Wnt-3a can reduce the exacerbated TNF-α levels through an autoregulatory feedback mechanism involving increased Fzd-1 receptors and activation of the Wnt/β-catenin pathway (Pereira et al., 2009; Neumann et al., 2010; Schaale et al., 2011). Additionally, Wnt-3a also promotes the expression of Arginase 1 in *M. tuberculosis-*infected macrophages, which has been associated with the anti-inflammatory M2 phenotype (Neumann et al., 2010).

Especially, crosstalk with inflammatory and oxidative stress pathways for the modulation of immune responses now highlights Wnt signaling as a critical modulator of M1/M2 pro/anti-inflammatory glial phenotype via both autocrine and paracrine effects (Chong and Maiese, 2007; Chong et al., 2010) (Figure 2). Work from our laboratory showed that during the acute mDA degeneration phase resulting from exposure to MPTP, microglia switches to M1 activated phenotype associated with up-regulated expression of NFκB and release of TNF-α and IL-1β cytokines, the up-regulation of PHOX-derived ROS and iNOS-derived NO and RNS, alltogether contributing to the acute loss of mDA cell bodies (L'Episcopo et al., 2011a; Figure 2). Another actor in glial activation cycle is represented by activation of the proinflammatory GSK-3β, leading to a vicious cycle of microglial activation (Jope et al., 2007; Beurel et al., 2010; Beurel, 2011; L'Episcopo et al., 2011a,b, 2016; Marchetti et al., 2013). Hence, NFκB and the Wnt/β-catenin pathway interact to differentially regulate inflammation: in a “*Wnt off”* condition, the activation GSK-3β positively regulates NFκB by targeting IkB (i.e., the major inhibitor of NFκB) to proteasomal degradation, which results in NFκB nuclear translocation and the induction of a proinflammatory genetic cascade, finally exacerbating microglia M1 phenotype (see Beurel et al., 2010; Neumann et al., 2010; Beurel, 2011). By contrast, in the “*Wnt On*” condition, cytosolic β-catenin accumulation can form a complex with the p50 subunit of NFκB, resulting in the prevention NF-kB transcriptional activity with consequent switch to the M2 microglia phenotype and downregulation of inflammation (Figure 2).

It seems important to note that the harmful M1 phenotype can itself promote an intrinsic Wnt/β-catenin rescue program both in neurons and glia. Hence, through glial expression of specific chemokines, such as CCL3, CXCl10, and CXCL11, astrocyte-derived Wnt1 is significantly up-modulated, both a mRNA and protein levels, and a progressive time-dependent neurorepair of nigrostriatal DA neurons and downregulation ofinflammation is observed (L'Episcopo et al., 2011a). Then, via astrocyte-microglia crosstalk and the release of Wnt1-like proteins in astrocytes, the resulting Wnt/β-catenin activation in microglial cells can inhibit GSK-3β activation, resulting in a downregulation of proinflammatory mediators (Chong and Maiese, 2007; Maiese et al., 2008; L'Episcopo et al., 2011a,b, 2013, 2014b; Schaale et al., 2011; Wang et al., 2012; Marchetti and Pluchino, 2013; Figure 2). In fact, the pharmacologic antagonism of GSK-3β restrain inflammatory microglial activation via the inhibition of proinflammatory cytokines through interactions at the level of NFκB (Beurel et al., 2010; Beurel, 2011; L'Episcopo et al., 2011a,b; Marchetti et al., 2013; Figure 2).

All together, an exaggerated microglial pro-inflammatory M1 status as observed with age and MPTP exposure, can impair astrocyte anti-inflammatory functions and mDA neurorescue (L'Episcopo et al., 2011a,b,c), via inhibition of Wnt1 expression and downregulation of anti-oxidant/anti-inflammatory cytoprotective proteins in astrocytes (L'Episcopo et al., 2013; Marchetti et al., 2013; Figures 1, 2).

## Astrocyte-microglia crosstalk and neural/stem progenitor cell (NSC) plasticity: Wnt signaling and inflammatory pathways shape the SVZ response to advancing age and PD

With age, the brain homeostatic and regenerative capacities progressively decline, at least in part as a result of a reduced tissue-specific self-adaptive potential and an impairment and/or a dysregulation of stem cell activity. Hence, a common hallmark in a number of age-dependent neurodegenerative diseases appears to be an alteration of adult neurogenesis (Curtis et al., 2007; He and Shen, 2009; Winner and Winkler, 2015). In mammals, one area where neuroblasts that give rise to adult-born neurons are generated is the subventricular zone (SVZ) (Lim and Alvarez-Buylla, 1999; Alvarez-Buylla et al., 2001; Kazanis, 2009; Ernst et al., 2014). In PD, a number of studies reported an impairment of the SVZ, where loss of the neurotransmitter dopamine, from mDA cell bodies innervating Type C cells in the SVZ, was causally related to the decreased neurogenic potential (Baker et al., 2004; Höglinger et al., 2004, 2012; Freundlieb et al., 2006; Borta and Höglinger, 2007; O'Keeffe et al., 2009a; Lennington et al., 2011). In addition, certain dopamine agonist therapies were reported to rescue NSC proliferation in PD (O'Keeffe et al., 2009a,b; Winner et al., 2009).

Notably, within the SVZ microenvironment (i.e., the “stem cell niche”), NSCs are in close contact with astroglial cells that modulate stem cell proliferation, migration and/or neuron differentiation, through the release of a panel of factors including morphogens, growth/neurotrophic factors and immunoregulatory molecules, thus implicating their active participation in NSC homeostatic regulation (Lim and Alvarez-Buylla, 1999; Alvarez-Buylla et al., 2001). Amongst others, Wnts are important modulators of adult neurogenesis, and *Wnt/*β*-catenin* is a vital pathway regulating self-renewal and differentiation of neural stem progenitor cells, NSCs (Adachi et al., 2007; Kalani et al., 2008; Kuwabara et al., 2009; Zhang L. et al., 2011; Shruster et al., 2012). Of special importance, inflammatory mechanisms both at the CNS and peripheral levels play an important role in the modulation of neurogenesis in the adult, aged and injured brain (Ekdahl et al., 2003, 2009; Jakubs et al., 2008; Pluchino et al., 2008; Thored et al., 2009; Martino et al., 2011; Tepavcević et al., 2011; Villeda et al., 2011; Cusimano et al., 2012; Ekdhal, 2012; Kokaia et al., 2012; L'Episcopo et al., 2012; Wallenquist et al., 2012; Wadhwa et al., 2017).

Hence, work in our laboratory focused on the potential for inflammation and astrocyte-microglia crosstalk to modulate the SVZ neurogenic niche, which is bordered by the corpus striatum. Here, NSC proliferative and neuron differentiation potential were monitored, both *in vivo* and *ex vivo*, as a function of aging and PD-induced morphological and functional changes of the striatal astroglial cell compartment. Additionally, we addressed the potential role of Wnt signaling in the neuroinflammatory regulation of SVZ neurogenesis. We thus uncovered that the Wnt/β-catenin signaling pathway is involved in the regulation of adult neurogenesis with advancing age and inflammation, and suggested crosstalk between inflammatory and *Wnt/*β*-catenin* signaling components (L'Episcopo et al., 2012). *In vivo* experiments showed an inverse correlation between the SVZ-neurogenic impairment of MPTP mice with the M1 glial activation status in striatum, with a maximal NSC inhibition corresponding to greatest microglia activation, as evidenced by increased of striatal iNOS, TNF-α, and IL-1β expression both at a mRNA and protein levels (L'Episcopo et al., 2012, 2013). These effects were associated to a marked β-catenin downregulation in the SVZ, in face of up-regulated levels of active pGSK-3β, reduced NSC proliferation and neuron differentiation (L'Episcopo et al., 2012, 2013). The observed up-regulation of active *pGSK-3*β in the face of β*-catenin* depletion in SVZ after MPTP exposure shown *in vivo*, was further supported both in *ex vivo* and *in vitro* experiments, further implicating disruption of β*-catenin* signaling in SVZ-NSC of MPTP mice (L'Episcopo et al., 2012, 2013).

*In vitro* studies using different coculture systems between young/aged glia with young/aged NSCs, and in the absence or the presence of MPTP/MPP^+^, next indicated that young M2-microglia increased NSC neurogenic potential, but upon MPP^+^ exposure, microglia shifted to the activated M1 phenotype and released high levels of pro-inflammatory mediators, inhibiting NSC proliferation, neuron differentiation and β-catenin expression, thus underscoring crosstalk between inflammatory and *Wnt/*β*-catenin* signaling components (L'Episcopo et al., 2011a, 2012, 2013). Importantly, astrocyte-microglia crosstalk was also shown to determine a further level of glial regulation of NSC neurogenic potential, as young astrocytes exposed to aged microglia fail to express Wnt1 and were no longer capable to promote NSC proliferation (L'Episcopo et al., 2013), suggesting that M1 phenotype sharply inhibits astrocyte proneurogenic capacities also via Wnt1 inhibition.

Interestingly, treatment with NO-flurbi of aged MPTP mice had the potential to rescue aging-induced SVZ impairment by a switch of the M1 harmful phenotype. This NO-flurbi-induced mitigation of the inflammatory SVZ microenvironment protected NSCs against mitochondrial impairment and cell death, and promoted proliferation and neurogenesis in the SVZ, which associated to a substantial striatal DA reinnervation both in young and aged mice MPTP mice, possibly resulting from NO-flurbi induced rescue of mDA neuronal cell bodies in the SN (L'Episcopo et al., 2013).

We then looked at one key factor involved in the mechanism by which cells combat oxidative stress and inflammation, the Nrf2-pathway, recognized to participate to nigrostriatal neuroprotection (Chen et al., 2009). Interestingly, we found that the Nrf2-antioxidant system was markedly impaired in SVZ astrocytes of aging mice, as a result of disrupted microglia-astrocyte crosstalk. This impairment, in turn, resulted in a failure of SVZ to adapt to the changing oxidative and inflammatory milieu of the aged SVZ niche (“the first hit”). Next, exposure to the PD neurotoxin (“the second hit”) in aging mice, further inhibited SVZ neurogenic potential (Figure 3). Interestingly, aged microglial inhibitory effects on NSCs proliferation and neuron formation was shown to rely on the *PI3K (phosphatidylinositol3-kinase)/Akt*-pathway, and with the intermediacy of the *Wnt/*β*-catenin* signaling cascade (L'Episcopo et al., 2013). Hence modulating *PI3K/Akt* and the *Wnt/Fzd/*β*-catenin* signaling cascades, was capable to switch on or off the activation of *GSK-3*β in SVZ-NSCs. Notably, NO-flurbi induced reversal of aging-induced SVZ impairment also associated to normalization of these age-related changes in Nrf2 and Wnt/β-catenin pathways (Figure 3), and significantly counteracted MPTP neurotoxic effects at striatal and SN levels.

Together, reactive astrocytes and microglia play a prominent role in the remodeling of the SVZ niche of PD rodents. Interestingly, glia-NSC interactions are in part regulated by crosstalk between inflammatory and Wnt/β-catenin signaling cascades. While further studies are clearly needed to address the causal relationship between the reversal of SVZ impairment and nigrostriatal neurorepair in aged-MPTP mice, such inflammatory modulation of SVZ neurogenesis herein described appears of special interest in light of accumulating evidence documenting that mitigating the inflammatory status, improving the neuronal microenvironment, and promoting mitochondrial function all together may represent a window of opportunity for therapeutic strategies aimed at upregulating endogenous neurogenesis, to favor the integration or survival of new neurons, to incite neurorepair, and/or to ameliorate some cognitive functions (Ehninger et al., 2011; L'Episcopo et al., 2012, 2014b; Rueger et al., 2012; Sakata et al., 2012; Vukovic et al., 2012; Wallenquist et al., 2012; Marchetti and Pluchino, 2013; Radad et al., 2017; Wadhwa et al., 2017; Yang et al., 2017).

## Genetic mutations, inflammation and mDA neurodegeneration: mRNAs/miRNAs and Wnt signaling interplay

Finally, the crucial link between inflammation and PD is further exemplified by the fact that key PD-associated genes, such as α-Syn (SNCA), PARK2, deglycase (DJ-1), leucine-rich repeat kinase 2 (LRRK2), and glucocerebrosidase (GBA) are all expressed in immune cells, suggesting their potential to modulate inflammation (Dzamko et al., 2015). Reciprocally, an increasing number of PD-related genes including LRRK2, VPS35, PINK1, UCHL-1, Parkin, ATP6AP2, and GBA modulate the canonical Wnt pathway (Berwick et al., 2017 and Refs. therein), further underlinying a critical Wnt/inflammatory connection in PD (Marchetti and Pluchino, 2013).

In addition a synergy between the genetic background and exposure to various neurotoxic or inflammatory challenges is recognized to promote a self-perpetuating cycle of microglial-mediated mDA neurotoxicity (Zhang et al., 2005; Gao and Hong, 2008, 2011; Marchetti et al., 2011; Gao et al., 2012; Lastres-Becker et al., 2012; Table 1). Notably, such feedforward cycle of chronic activation of microglia and chronic damage of mDA neurons are likely to play a decisive role for the severity of nigrostriatal DA lesion and the overall detrimental effects of SNpc neurons and consequently, their capacity for neurorepair.

A number of laboratories showed the harmful consequences of dysfunctional α-Syn coupled to the M1 pro-inflammatory phenotype, capable to potentiate each other and promote the progression of mDA neuron death (Gao et al., 2011; Harms et al., 2013; Sanchez-Guajardo et al., 2013). Notably, Lastres-Becker et al. (2012) reported that a dysfunctional anti-oxidant system in NRf2-deficient mice coupled to α-syn dysfunction in early-stage of PD can synergize together resulting in exacerbated inflammation, up-regulated protein aggregation, all together promoting increased neuronal death. Additionally, as reported by Frank-Cannon et al. (2008), Parkin (the product of the *PARK2* gene) deficiency, increases the vulnerability of mDA neurons to various risk factors including inflammation-dependent degeneration. Another important connection is the one between LRRK2 mutation and the activation of M1 proinflammatory phenotype (Gillardon et al., 2012), acting in synergy to amplify mDA neurotoxicity. By contrast, when LRRK2 is inhibited, this in turn reduces the production of microglial harmful mediators and reverses mDA neurotoxicity (Kim et al., 2012; Moehle et al., 2012; Lee et al., 2017). Notably, a robust LRRK2 expression is present in immune cells, including peripheral monocytes and macrophages, and in primary microglia (Dzamko et al., 2015). Of interest, peripheral inflammation appears greater in a percentage of subjects carrying LRRK2-G2019S mutation, with the cytokine IL-1β discriminating asymptomatic LRRK2-G2019S carriers from controls (Dzamko et al., 2017). Furthermore, the expression of LRRK2 is modulated by immune cell-specific signals, like IFNγ and toll-like receptor (TLR) agonists (see Moehle et al., 2012) thereby reinforcing the LRRK2/immunological link.

Notably, LRRK2 binds three central Wnt signaling components (Sancho et al., 2009; Berwick and Harvey, 2012a), while loss of LRRK2 and mutations of *LRRK2* are linked to Wnt signaling (Sancho et al., 2009; Berwick and Harvey, 2012a,b, 2014; Berwick et al., 2017). Hence, pathogenic *PARK8* mutations impact upon the activity of the canonical Wnt pathway (Berwick and Harvey, 2012a). Recent evidence indicates that in the context of canonical Wnt signaling, pathological LRRK2 mutations are gain-of function, enhancing the repression of β-catenin mediated by LRRK2, thus inhibitng *canonical* Wnt/β-catenin signaling (Berwick et al., 2017). Such connection between LRRK2 and Wnt cascades in PD support the growing body of studies highlighting dysregulated Wnt signaling in PD (see Harvey and Marchetti, 2014 and chapters therein).

*PARK17* encodes the vacuolar protein sortin 35 homolog gene, VPS3, and its mutation is linked to autosomal dominant late-onset PD, with an involvement in iron up-take and Wnt/β-catenin signaling (Deng et al., 2013). Of note iron together with other risk factors, such as exposure to paraquat, may interact to aggravate neuroinflammation and age-dependent mDA neuron death (Peng et al., 2007).

Further compelling evidences from the last few years implicate certain miRNAs in the counter-regulation of microglia M1 phenotype associated to robust activation (Table 2). For example the group of Prajapati in 2015 found that TNF-α was able to both trigger cell death and sensitize to apoptosis the DA cell line SH-SY5Y, in the presence of different PD neurotoxins—such as MPP+, 6-OHDA and Rotenone—via miRNA deregulation (Prajapati et al., 2015). Following the treatment with TNF-α, 9 miRNAs were found upregulated (let-7b, let-7g, miR-103, miR-155, miR-16-5p, miR-17, miR-204, miR-27, and miR-98) and 7 downregulated (let-7a, miR-128, miR-145, miR-181a, miR23a, miR-23b, and miR-320a). Interestingly, the upregulated miRNAs were predicted to target mRNAs involved in both neuronal-specific pathways (i.e., neuronal differentiation, axonal guidance and nerve projection development) and mitochondrial respiratory subunits. In particular, the authors demonstrated that, in the presence of TNF-α, both miR-155 and miR-27 were able to downregulate ATP5G3, a subunit of F1-ATP synthase. This study strongly supports the role of TNF-α as a critical regulator of miRNAs targeting mitochondrial functions, which in turn may cause DA neuronal loss (L'Episcopo et al., 2010a,b, 2011c; Prajapati et al., 2015).

**Table 2:** M1 pro-inflammatory phenotype and miRNA dysregulation in PD.

**miRNAs**	**Expression levels**		**Outcomes**	**References**
let-7b, let-7g, miR-103, miR-155, miR-16-5p, miR-17, miR-204, miR-27, miR-98	↑	**Upregulation** following TNF-α treatment in SH-SY5Y cells		Prajapati et al., 2015
let-7a, miR-128, miR-145, miR-181a, miR23a, miR-23b, miR-320°	↓	**Downregulation** following TNF-α treatment in SH-SY5Y cells		
miR-155, miR-27	↑	**Upregulation** following TNF-α treatment in SH-SY5Y cells	ATP5G3 (F1-ATP synthase subunit) downregulation in mitochondria of SH-SY5Y cells	
miR-155	↓	**Downregulation** following *antago-miR-155* administration in TNF-α-treated SH-SY5Y cells	Increased SH-SY5Y cells survival following TNF-α treatment	
miR-155	↑	**Upregulation** following LPS, IFN-γ or TNF-α treatments in THP-1 cells	Downregulation of FADD, SOC1, IKK, IL13Rα1 and SMAD2	Louafi et al., 2010; Liu and Abraham, 2013; Ponomarev et al., 2013; Yang et al., 2015
	↓	**Downregulation** following *antago-miR-155* administration in atherosclerosis mouse model	Downregulation of TNF-α, IL-1β, CCL2, CCL4, and CCL7 secretion in serum and vascular tissues	Yang et al., 2015
	↑	**Upregulation** in PD mice overexpressing α-SYN	Inflammatory response to α-SYN fibrils and reactive microgliosis	Thome et al., 2016
miR-7	↓	**Downregulation** in neurons of MPTP-treated mice	α-SYN upregulation	Junn et al., 2009; Zhou et al., 2016
	↑	**Upregulation** following *miR-7 mimic* injection in MPTP-treated mice	Downregulation of α-SYN and downregulation of NRLP3 in DA neurons with suppression of inflammasome-mediated neuroinflammation and attenuated DA neurodegeneration	
	↓	**Downregulation** following *antago-miR-7* administration in MSU or ATP treated BV2 cells	Upregulation of NLRP3 expression and aggravated inflammasome activation *in vitro*	
miR-135b	↓	**Downregulation** in MPP+-treated SH-SY5Y cells	GSK3β upregulation^**§**^	Wang et al., 2007; L'Episcopo et al., 2011a,b; Zhang et al., 2017
	↑	**Upregulation** following *miR-135b mimic* administration in SH-SY5Y cells	GSK3β downregulation, TNF-α and IL-1β reduction, MPP+-induced apoptosis rescue^**§**^	
miR-7116-5p	↓	**Downregulation** in microglia of MPTP-treated mice	miR-7116-5p directly targets and inhibits TNF-α expression. In MPTP mice miR-7116-5p is downregulated, consequently TNF-α production is boosted	He et al., 2017
	↑	**Upregulation** following *lentiviral-mediated miR-7116-5b overexpression* in microglia of MPTP-treated mice	Downregulation of TNF-α, reduction of TNF-α-mediated inflammatory activation and prevention of DAergic neuronal loss	

§ *Wnt/β-Catenin dysregulation in the reported conditions*.

Notably, miR-155 was previously shown to be involved in the regulation of inflammatory processes. The induction of miR-155 (via LPS, IFN-γ, and TNF-α) is able to target key regulators of inflammation, such as FADD, SOC1, IKK, IL13Rα1, and SMAD2, while miR-155 inhibition results in the upregulation of the proinflammatory molecules IL-1β, IL-6, TNF-α, and iNOS (Louafi et al., 2010; Liu and Abraham, 2013; Ponomarev et al., 2013; Yang et al., 2015).

The relevance of miR-155 in PD was confirmed in 2016 by Thome and colleagues that observed miR-155 upregulation in a PD mouse model overexpressing α-SYN. They demonstrated that miR-155 is crucial to mediate the inflammatory response to α-SYN fibrils, responsible of reactive microgliosis and accounting for the loss of DA neurons, triggered by the overexpression of α-SYN (Thome et al., 2016).

Other miRNAs are recently emerging as important regulators of M1 microglial pro-inflammatory phenotype, such as miR-7, previously reported to target α-SYN in DA neurons (Junn et al., 2009). In 2016 miR-7 was demonstrated to directly target microglial nod-like receptor protein 3 gene (NRLP3), suppressing inflammasome-mediated neuroinflammation and thus suggesting a potential therapeutic role of this miRNAs in the context of PD (Zhou et al., 2016).

There are also interesting clues linking Wnt/β-catenin pathway to miRNA-modulation of DA neuronal survival and inflammation (Table 2). In fact, the role of miR-135b as GSK3β regulator was recently investigated in MPP^+^-treated SH-SY5Y cells (Zhang et al., 2017). The specific pharmacological inhibition of GSK3β reversed MPTP-induced neuron injury and also improves MPTP-induced behavioral impairment (Wang et al., 2007; L'Episcopo et al., 2011a,b). Interestingly, miR-135b was reduced in face of GSK3β upregulation in MPP^+^-treated cells, in a dose- and a time-dependent manner. Importantly, the overexpression of miR-135b was able to directly target GSK3β, and to reduce the levels of pro-inflammatory cytokines TNF-α and IL-1β, thus rescuing the MPP^+^-induced apoptosis (Zhang et al., 2017).

The same year, also miR-7116-5p was suggested to be a key player in neuroinflammation. Specifically in microglia of an MPTP mouse model, miR-7116-5p was found to be downregulated, while TNF-α increased. This miRNA was demonstrated to directly target TNF-α transcript, thus reducing TNF-α-mediated inflammatory activation and finally preventing DAergic neuronal loss in MPTP mice (He et al., 2017).

Together, gene-environment interactions crucially impact in switching microglia status to the M1 neuron destructive phenotype, with the contribution of both mRNAs and miRNAs, and Wnt/β-catenin signaling interplay.

## Concluding remarks and future perspectives

In this work we have highlighted the evidences documenting a major role of gene-environment interactions directing the polarization of microglia toward an harmful M1 phenotype, that may predispose the brain to reach a critical threshold of inflammation, triggering a self-perpetuating cycle of inflammation and neuronal death. Especially, we pinpointed the role of Wnt signaling in each of the steps involved in both the neuroprotective/destructive glial-mediated neuronal outcome in PD.

Aging is a critical period for the vulnerability to PD. Importantly, aging reduces the degree of DAergic neuron plasticity, diminishes mDA neuron adaptive capacity, exacerbates inflammation and impair neurogenesis, at least in part via a dysfunction Wnt/β-catenin signaling and the crosstalk with inflammatory pathways. The inflammatory involvement in the regulation of adult neurogenesis suggest that harnessing inflammatory responses through targeted modulation of innate immunity during the pre-motor phase of PD may have potential therapeutic implications to incite endogenous neurogenesis and neurorepair in PD. Finally, aging, inflammation and major genetic mutations, together with a set of recently uncovered inflammation-dependent miRNA, all together impact on Wnt/β-catenin signaling pathway, with potential consequences for PD degeneration.

All together, unraveling the complex molecular circuity linking key molecular genetic and environmental drivers in PD with microglia polarization will permit to identify new drugable targets for the cure of PD.

## Author contributions

All authors listed have made a substantial, direct and intellectual contribution to the work, and approved it for publication.

### Conflict of interest statement

The authors declare that the research was conducted in the absence of any commercial or financial relationships that could be construed as a potential conflict of interest.

## References

[B1] Abou-Sleiman P. M. Muqit M. M. Wood N. W. (2006). Expanding insights of mitochondrial dysfunction in Parkinson's disease. Nat. Rev. Neurosci. 7, 207–219. 10.1038/nrn1868 16495942

[B2] Adachi K. Mirzadeh Z. Sakaguchi M. Yamashita T. Nikolcheva T. Gotoh Y.. (2007). β-catenin signaling promotesproliferation of progenitor cells in the adult mouse subventricular zone. Stem Cells 25, 2827–2836. 10.1634/stemcells.2007-0177 17673525

[B3] Alvarez-Buylla A. García-Verdugo J. M. Tramontin A. D. (2001). A unified hypothesis on the lineage of neural stem cells. Nat. Rev. Neurosci. 2, 287–293. 10.1038/35067582 11283751

[B4] Angers S. Moon R. T. (2009). Proximal events in Wnt signal transduction. Nat. Rev. Mol. Cell Biol. 10, 468–477. 10.1038/nrm2717 19536106

[B5] Arenas E. (2014). Wnt signaling in midbrain dopaminergic neuron development and regenerative medicine for Parkinson's disease. J. Mol. Cell Biol. 6, 42–53. 10.1093/jmcb/mju001 24431302

[B6] Baba Y. Kuroiwa A. Uitti R. Wszolek J. Yamada T. (2005). Alterations of T-lymphocyte populations in Parkinson's disease. Parkinson. Relat. Disord. 11, 493–498. 10.1016/j.parkreldis.2005.07.005 16154792

[B7] Baker S. A. Baker K. A. Hagg T. (2004). Dopaminergic nigrostriatal projections regulate neural precursor cell proliferation in the adult mouse subventricular zone. Eur. J. Neurosci. 20, 575–579. 10.1111/j.1460-9568.2004.03486.x 15233767

[B8] Barcia C. Ros C. M. Ros-Bernal F. Gómez A. Annese V. Carrillo-de Sauvage M. A.. (2013). Persistent phagocytic characteristics of microglia in the substantia nigra of long-term Parkinsonian macaques. J. Neuroimmunol. 261, 60–66. 10.1016/j.jneuroim.2013.05.001 23759319

[B9] Bartel D. P. (2004). MicroRNAs: genomics, biogenesis, mechanism, and function. Cell 116, 281–297. 10.1016/S0092-8674(04)00045-5 14744438

[B10] Bélanger M. Magistretti P. J. (2009). The role of astroglia in neuroprotection. Dialog. Clin. Neurosci. 11, 281–295. 19877496 10.31887/DCNS.2009.11.3/mbelanger PMC3181926

[B11] Bendor J. T. Logan T. P. Edwards R. H. (2013). The function of α-synuclein. Neuron 79, 1044–1066. 10.1016/j.neuron.2013.09.004 24050397 PMC3866954

[B12] Berwick D. C. Harvey K. (2012a). LRRK2 functions as a Wnt signaling scaffold, bridging cytosolic proteins and membrane-localized LRP6. Hum. Mol. Genet. 21, 4966–4979. 10.1093/hmg/dds342 22899650 PMC3709196

[B13] Berwick D. C. Harvey K. (2012b). The importance of Wnt signalling for neurodegeneration in Parkinson's disease. Biochem. Soc. Trans. 40, 1123–1128. 10.1042/BST20120122 22988876

[B14] Berwick D. C. Harvey K. (2014). The regulation and deregulation of Wnt signalling by PARK genes in health and disease. J. Mol. Cell Biol. 6, 3–12. 10.1093/jmcb/mjt037 24115276 PMC4344548

[B15] Berwick D. C. Javaheri B. Wetzel A. Hopkinson M. Nixon-Abell J. Grannò S.. (2017). Pathogenic LRRK2 variants are gain-of-function mutations that enhance LRRK2-mediated repression of β-catenin signaling. Mol. Neurodegener. 12:9. 10.1186/s13024-017-0153-4 28103901 PMC5248453

[B16] Beurel E. (2011). Regulation by glycogen synthase kinase-3 of inflammation and T cells in CNS diseases. Front. Mol. Neurosci. 4:18. 10.3389/fnmol.2011.00018 21941466 PMC3171068

[B17] Beurel E. Michalek S. M. Jope R. S. (2010). Innate and adaptive immune responses regulated by glycogen synthase kinase-3(GSK3). Trends Immunol. 31, 24–31. 10.1016/j.it.2009.09.007 19836308 PMC2818223

[B18] Bian S. Sun T. (2011). Functions of noncoding RNAs in neural development and neurological diseases. Mol. Neurobiol. 44, 359–373. 10.1007/s12035-011-8211-3 21969146 PMC3246283

[B19] Bilic J. Huang Y.-L. Davidson G. Zimmermann T. Cruciat C.-M. Bienz M.. (2007). Wnt induces LRP6 signalosomes and promotes dishevelled-dependent LRP6 phosphorylation. Science 316, 1619–1622. 10.1126/science.1137065 17569865

[B20] Blandini F. Armentero M. T. (2012). Animal models of Parkinson's disease. FEBS J. 279, 1156–1166. 10.1111/j.1742-4658.2012.08491.x 22251459

[B21] Boger H. A. Granholm A. C. McGinty J. F. Middaugh L. D. (2010). A dual-hit animal model for age-related parkinsonism. Prog. Neurobiol. 90, 217–229. 10.1016/j.pneurobio.2009.10.013 19853012 PMC3991553

[B22] Borta A. Höglinger G. U. (2007). Dopamine and adult neurogenesis. J. Neurochem. 100, 587–595. 10.1111/j.1471-4159.2006.04241.x 17101030

[B23] Bové J. Perier C. (2012). Neurotoxin-based models of Parkinson's disease. Neuroscience 211, 51–76. 10.1016/j.neuroscience.2011.10.057 22108613

[B24] Bovolenta P. Esteve P. Ruiz J. M. Cisneros E. Lopez-Rios J. (2008). Beyond Wnt inhibition: new functions of secreted Frizzled-related proteins in development and disease. J Cell Sci. 121, 737–746. 10.1242/jcs.026096 18322270

[B25] Brochard V. Combadière B. Pigent A. Laouar Y. Perrin A. Beray-Berthat V.. (2009). Infiltration of CD4+ lymphocytes into the brain contributes to neurodegeneration in a mouse model of Parkinson disease. J. Clin. Invest. 119, 182–192. 10.1172/JCI36470 19104149 PMC2613467

[B26] Cadigan K. M. Waterman M. L. (2012). TCF/LEFs and Wnt signaling in the nucleus. Cold Spring Harb. Perspect. Biol. 4:a007906. 10.1101/cshperspect.a007906 23024173 PMC3536346

[B27] Cannon J. R. Greenamyre J. T. (2013). Gene-environment interactions in Parkinson's disease: specific evidence in humans and mammalian models. Neurobiol. Dis. 57, 38–46. 10.1016/j.nbd.2012.06.025 22776331 PMC3815566

[B28] Chen G. Bower K. A. Ma C. Fang S. Thiele C. J. Luo J. (2004). Glycogen synthase kinase 3beta (GSK3beta) mediates 6-hydroxy dopamine-induced neuronal death. FASEB J. 18, 1162–1164. 10.1096/fj.04-1551fje 15132987

[B29] Chen H. Jacobs E. J. Schwarzschild M. A. McCullough M. L. Calle E. E. Thun M. J. (2005). Nonsteroidal anti-inflammatory drug use and the risk for Parkinson's disease. Ann. Neurol. 58, 963–967. 10.1002/ana.20682 16240369

[B30] Chen H. Zhang S. M. Herman M. A. Schwarzschild M. A. Willett W. C. Colditz G. A. (2003). Nonsteroidal anti-inflammatory drugs and the risk of Parkinson's disease. Arch. Neurol. 60, 1059–1064. 10.1001/archneur.60.8.1059 12925360

[B31] Chen P. C. Vargas M. R. Pani A. K. Smeyne R. J. Johnson D. A. Kan Y. W.. (2009). Nrf2-mediated neuroprotection in the MPTP mouse model of Parkinson's disease: Critical role for the astrocyte. Proc. Natl. Acad. Sci. U.S.A. 106, 2933–2938. 10.1073/pnas.0813361106 19196989 PMC2650368

[B32] Chen Y. Qi B. Xu W. Ma B. Li L. Chen Q. (2015). Clinical correlation of peripheral CD4^+^-ccell sub-sets, their imbalance and Parkinson's disease. Mol. Med. Rep. 12, 6105–6111. 26239429 10.3892/mmr.2015.4136

[B33] Chong Z. Z. Maiese K. (2007). Cellular demise and inflammatory microglial activation during beta-amyloid toxicity are governed by Wnt1 and canonical signalling pathways. Cell Signal. 19, 1150–1162. 10.1016/j.cellsig.2006.12.009 17289346 PMC1913492

[B34] Chong Z. Z. Shang Y. C. Hou J. Maiese K. (2010). Wnt1 neuroprotection translates into improved neurological function during oxidant stress and cerebral ischemia through AKT1 and mitochondrial apoptotic pathways. Oxidat. Med Cell. Long. 3, 153–165. 10.4161/oxim.3.2.11758 20716939 PMC2952099

[B35] Ciani L. Salinas P. C. (2005). WNTs in the vertebrate nervous system: From patterning to neuronal connectivity. Nat. Rev. Neurosi. 6, 351–362. 10.1038/nrn1665 15832199

[B36] Clevers H. (2006). Wnt/beta-catenin signaling in development and disease. Cell 127, 469–480. 10.1016/j.cell.2006.10.018 17081971

[B37] Clevers H. Nusse R. (2012). Wnt/beta-catenin signaling and disease. Cell 149, 1192–1205. 10.1016/j.cell.2012.05.012 22682243

[B38] Codolo G. Plotegher N. Pozzobon T. Brucale M. Tessari I. Bubacco L.. (2013). Triggering of inflammasome by aggregated α-synuclein, an inflammatory response in synucleinopathies. PLoS ONE 8:e55375. 10.1371/journal.pone.0055375 23383169 PMC3561263

[B39] Collier T. J. Lipton J. Daley B. F. Palfi S. Chu Y. Sortwell C.. (2007). Aging-related changes in the nigrostriatal dopamine system and the response to MPTP in nonhuman primates: diminished compensatory mechanisms as a prelude to parkinsonism. Neurobiol. Dis. 26, 56–65. 10.1016/j.nbd.2006.11.013 17254792 PMC1899875

[B40] Collins L. M. Toulouse A. Connor T. J. Nolan Y. M. (2012). Contributions of central and systemic inflammation to the pathophysiology of Parkinson's disease. Neuropharmacology 62, 2154–2168. 10.1016/j.neuropharm.2012.01.028 22361232

[B41] Cunningham C. Wilcockson D. C. Campion S. Lunnon K. Perry V. H. (2005). Central and systemic endotoxin challenges exacerbate the local inflammatory response and increase neuronal death during chronic neurodegeneration. J. Neurosci. 25, 9275–9284. 10.1523/JNEUROSCI.2614-05.2005 16207887 PMC6725757

[B42] Curtis M. A. Faull R. L. M. Eriksson P. S. (2007). The effect of neurodegenerative diseases on the subventricular zone. Nat. Rev. Neurosci. 8, 712–723. 10.1038/nrn2216 17704813

[B43] Cusimano M. Biziato D. Brambilla E. Donegà M. Alfaro-Cervello C. Snider S.. (2012). Transplanted neural stem/precursor cells instruct phagocytes and reduce secondary tissue damage in the injured spinal cord. Brain 135, 447–460. 10.1093/brain/awr339 22271661 PMC3558737

[B44] Damani M. R. Zhao L. Fontainhas A. M. Amaral J. Fariss R. N. (2010). Age-related alterations in the dynamic behavior of microglia. Aging Cell 10, 263–276. 10.1111/j.1474-9726.2010.00660.x 21108733 PMC3056927

[B45] de la Fuente-Fernández R. Schulzer M. Kuramoto L. Cragg J. Ramachandiran N. Au W. L.. (2011). Age-specific progression of nigrostriatal dysfunction in Parkinson's disease. Ann. Neurol. 69, 803–810. 10.1002/ana.22284 21246604

[B46] Deleidi M. Hallett P. J. Koprich J. B. Chung C. Y. Isacson O. (2010). The Toll-like receptor-3 agonist polyinosinic:polycytidylic acid triggers nigrostriatal dopaminergic degeneration. J. Neurosci. 30, 16091–16101. 10.1523/JNEUROSCI.2400-10.2010 21123556 PMC3075577

[B47] Deng H. Gao K. Jankovic J. (2013). The VPS35 gene and Parkinson's disease. Mov Disord. 28, 569–575. 10.1002/mds.25430 23536430

[B48] Depboylu C. Stricker S. Ghobril J. P. Oertel W. H. Priller J. Höglinger G. U. (2012). Brain-resident microglia predominate over infiltrating myeloid cells in activation, phagocytosis and interaction with T-lymphocytes in the MPTP mouse model of Parkinson disease. Exp. Neurol. 238, 183–191. 10.1016/j.expneurol.2012.08.020 22964486

[B49] Di Monte D. A. Langston J. W. (1995). Idiopathic and 1-methyl-4phenyl-1,2,3,6-tetrahydropyridine (MPTP)-induced Parkinsonism, in Neuroglia, Chapter 65, eds Kettenmann H. Ransom B. R. (New York, NY: Oxford University Press), 997–989.

[B50] Di Monte D. A. Lavasani M. Manning-Bog A. B. (2002). Environmental factors in Parkinson's disease. NeuroToxicology 23, 487–502. 10.1016/S0161-813X(02)00099-2 12428721

[B51] Duka T. Duka V. Joyce J. N. Joyce J. N. Sidhu A. (2009). α-Synuclein contributes to GSK-3β- catalyzed Tau phosphorylation in Parkinson's disease models. FASEB J. 23, 2820–2830. 10.1096/fj.08-120410 19369384 PMC2796901

[B52] Dzamko N. Geczy C. L. Halliday G. M. (2015). Inflammation is genetically implicated in Parkinson's disease. Neuroscience 302, 89–102. 10.1016/j.neuroscience.2014.10.028 25450953

[B53] Dzamko N. Gysbers A. Perera G. Bahar A. Shankar A. Gao J.. (2017). Toll-like receptor 2 is increased in neurons in Parkinson's disease brain and may contribute to alpha-synuclein pathology. Acta Neuropathol. 133, 303–319. 10.1007/s00401-016-1648-8 27888296 PMC5250664

[B54] Ehninger D. Wang L. P. Klempin F. Römer B. Kettenmann H. Kempermann G. (2011). Enriched environment and physical activity reduce microglia and influence the fate of NG2 cells in the amygdala of adult mice. Cell Tissue Res. 345, 69–86. 10.1007/s00441-011-1200-z 21688212 PMC3132349

[B55] Ekdahl C. T. Claasen J. H. Bonde S. Kokaia Z. Lindvall O. (2003). Inflammation is detrimental for neurogenesis in the adult brain. Proc. Natl. Acad. Sci. U.S.A. 100, 13632–13637. 10.1073/pnas.2234031100 14581618 PMC263865

[B56] Ekdahl C. T. Kokaia Z. Lindvall O. (2009). Brain inflammation and adult neurogenesis: the dual role of microglia. Neuroscience 158, 1021–1029. 10.1016/j.neuroscience.2008.06.052 18662748

[B57] Ekdhal C. T. (2012). Microglial activation: tuning and pruning adult neurogenesis. Front. Pharmacol. 3:41 10.3389/fphar.2012.00041 22408626 PMC3297835

[B58] Ernst A. Alkass K. Bernard S. Salehpour M. Perl S. Tisdale J.. (2014). Neurogenesis in the striatum of the adult human brain. Cell 156, 1072–1083. 10.1016/j.cell.2014.01.044 24561062

[B59] Esposito E. Di Matteo V. Benigno A. Pierucci M. Crescimanno G. Di Giovanni G. (2007). Non-steroidal anti-inflammatory drigs in parkinson's disease. Exp. Neurol. 205, 295–312. 10.1002/mds.20856 17433296

[B60] Ferreira M. Massano J. (2016). An updated review of Parkinson's disease genetics and clinico-pathological correlations. Acta Neurol. Scand. 135, 273–284. 10.1111/ane.12616 27273099

[B61] Fiorucci S. Antonelli E. (2006). NO-NSAIDs: from inflammatory mediators to clinical readouts. Inflam. Allergy Drug Targets 5, 121–131. 10.2174/187152806776383161 16613571

[B62] Flanary B. (2005). The role of microglial cellular senescence in the aging and Alzheimer diseased brain. Rejuvenat. Res. 8, 82–85. 10.1089/rej.2005.8.82 15929715

[B63] Flanary B. E. Sammons N. W. Nguyen C. Walker D. Streit W. J. (2007). Evidence that aging and amyloid promote microglial cell senescence. Rejuvenation Res. 10, 61–74. 10.1089/rej.2006.9096 17378753

[B64] Frank-Cannon T. C. Tran T. Ruhn K. A. Martinez T. N. Hong J. Marvin M.. (2008). Parkin deficiency increases vulnerability to inflammation-related nigral degeneration. J. Neurosci. 28, 10825–10834. 10.1523/JNEUROSCI.3001-08.2008 18945890 PMC2603252

[B65] Freundlieb N. François C. Tandé D. Ortel W. H. Hirsh E. C. Höglinger G. U. (2006). Dopaminergic subtantia nigra neurons project topographically organized to the subventricular zone and stimulate precursor cell proliferation in aged primates. J. Neurosci. 26, 2321–2325. 10.1523/JNEUROSCI.4859-05.2006 16495459 PMC6674815

[B66] Galli S. Lopes D. M. Ammari R. Kopra J. Millar S. E. Gibb A.. (2014). Deficient Wnt signalling triggers striatal synaptic degeneration and impaired motor behaviour in adult mice. Nat. Commun. 5:4992. 10.1038/ncomms5992 25318560 PMC4218967

[B67] Gao H. M. Hong J. S. (2008). Why neurodegenerative diseases are progressive: uncontrolled inflammation drives disease progression. Trends Immunol. 29, 357–365. 10.1016/j.it.2008.05.002 18599350 PMC4794280

[B68] Gao H. M. Hong J. S. (2011). Gene-environment interactions: key to unraveling the mystery of Parkinson's disease. Prog. Neurobiol. 94, 1–19. 10.1016/j.pneurobio.2011.03.005 21439347 PMC3098527

[B69] Gao H. M. Zhang F. Zhou H. Kam W. Wilson B. Hong J. S. (2011). Neuroinflammation and α-synuclein dysfunction potentiate each other, driving chronic progression of neurodegeneration in a mouse model of Parkinson's disease. Environ. Health Perspect. 119, 807–814. 10.1289/ehp.1003013 21245015 PMC3114815

[B70] Gao J. Nalls M. A. Shi M. Joubert B. R. Hernandez D. G. Huang X.. (2012). An exploratory analysis on gene-environment interactions for Parkinson disease. Neurobiol. Aging 33, 2528.e1–6. 10.1016/j.neurobiolaging.2012.06.007 22763023 PMC3419385

[B71] Gennuso F. Fernetti C. Tirolo C. Testa N. L'Episcopo F. Caniglia S. (2004). Bilirubin protects astrocytes from its own toxicity inducing up-regulation and translocation of multigrug resistance-associated protein 1 (Mrp 1). Proc. Natl. Acad. Sci. U.S.A. 101, 2470–2475. 10.1073/pnas.0308452100 14983033 PMC356974

[B72] Gerhard A. Pavese N. Hotton G. Turkheimer F. Es M. Hammers A. (2006). *In vivo* imaging of microglial activation with [11C](R)-PK11195 PET in idiopathic Parkinson's disease. Neurobiol. Dis. 21, 404–412. 10.1016/j.nbd.2005.08.002 16182554

[B73] Gillardon F. Schmid R. Draheim H. (2012). Parkinson's disease-linked leucine-rich repeat kinase 2(R1441G) mutation increases proinflammatory cytokine release from activated primary microglial cells and resultant neurotoxicity. Neuroscience 208, 41–48. 10.1016/j.neuroscience.2012.02.001 22342962

[B74] Glinka A. Dolde C. Kirsch N. Huang Y. L. Kazanskaya O. Ingelfinger D.. (2011). LGR4 and LGR5 are R-spondin receptors mediating Wnt/beta-catenin and Wnt/PCP signalling. EMBO Rep. 12, 1055–1061. 10.1038/embor.2011.175 21909076 PMC3185347

[B75] Godbout J. P. Chen J. Abraham J. Richwine A. F. Berg B. M. Kelley K. W. (2005). Exaggerated neuroinflammation and sickness behaviour in aged mice after activation of the peripheral innate immune system. FASEB J. 19, 1329–1331. 10.1096/fj.05-3776fje 15919760

[B76] Gordon M. D. Nusse R. (2006). Wnt signaling: multiple pathways, multiple receptors and multiple transcription factors. J. Biol. Chem. 281, 22429–22433. 10.1074/jbc.R600015200 16793760

[B77] Grimes C. A. Jope R. S. (2001). The multifaceted roles of glycogen synthase kinase 3beta in cellular signaling. Prog. Neurobiol. 65, 391–426. 10.1016/S0301-0082(01)00011-9 11527574

[B78] Halleskog C. Dijksterhuis J. P. Kilander M. B. Becerril-Ortega J. Villaescusa J. C. Lindgren E. (2012). Heterotrimeric G protein-dependent WNT-5A signaling to ERK1/2 mediates distinct aspects of microglia proinflammatory transformation. J. Neuroinflammation 30:111 10.1186/1742-2094-9-111 PMC3458933 22647544

[B79] Halleskog C. Mulder J. Dahlström J. Mackie K. Hortobágyi T. Tanila H.. (2011). WNT signaling in activated microglia is proinflammatory. Glia 59, 119–131. 10.1002/glia.21081 20967887 PMC3064522

[B80] Halleskog C. Schulte G. (2013a). WNT-3A and WNT-5A counteract lipopolysaccharide-induced pro-inflammatory changes in mouse primary microglia. Neurochem. 125, 803–808. 10.1111/jnc.12250 23534675

[B81] Halleskog C. Schulte G. (2013b). Pertussis toxin-sensitive heterotrimeric G(αi/o) proteins mediate WNT/β-catenin and WNT/ERK1/2 signaling in mouse primary microglia stimulated with purified WNT-3A. Cell Signal. 25, 822–828. 10.1016/j.cellsig.2012.12.006 23266471

[B82] Hamza T. H. Zabetian C. P. Tenesa A. Laederach A. Montimurro J. Yearout D.. (2010). Common genetic variation in the HLA region is associated with late-onset sporadic Parkinson's disease. Nat. Genet. 42, 781–785. 10.1038/ng.642 20711177 PMC2930111

[B83] Harms A. S. Cao S. Rowse A. L. Thome A. D. Li X. Mangieri L. R.. (2013). MHCII is required for α-synuclein-induced activation of microglia, CD4 T cell proliferation, and dopaminergic neurodegeneration. J. Neurosci. 33, 9592–9600. 10.1523/JNEUROSCI.5610-12.2013 23739956 PMC3903980

[B84] Harvey K. Marchetti B. (2014). Regulating Wnt signaling: a strategy to prevent neurodegeneration and induce regeneration. J. Mol. Cell Biol. 6, 1–2. 10.1093/jmcb/mju002 24549156

[B85] He P. Shen Y. (2009). Interruption of β-catenin signaling reduces neurogenesis in Alzheimer's disease. J. Neurosci. 29, 6545–6557. 10.1523/JNEUROSCI.0421-09.2009 19458225 PMC3618977

[B86] He Q. Wang Q. Yuan C. Wang T. (2017). Downregulation of miR-7116-5p in microglia by MPP(+) sensitizes TNF-α production to induce dopaminergic neuron damage. Glia 65, 1251–1263. 10.1002/glia.23153 28543680

[B87] Henry C. J. Huang Y. Wynne A. Godbout J. P. (2009). Peripheral lipopolysaccharide (LPS) challenge promotes microglial hyperactivity in aged mice that is associated with exaggerated induction of both pro-inflammatory IL-1β and anti-inflammatory IL-10 cytokines. Brain Behav. Immun. 23, 309–317. 10.1016/j.bbi.2008.09.002 18814846 PMC2692986

[B88] Hindle J. V. (2010). Ageing, neurodegeneration and Parkinson's disease. Age Ageing 39, 156–161. 10.1093/ageing/afp223 20051606

[B89] Hirsch E. C. Hunot S. (2009). Neuroinflammation in Parkinson's disease: a target for neuroprotection? Lancet Neurol. 8, 382–397. 10.1016/S1474-4422(09)70062-6 19296921

[B90] Hirsch E. C. Jenner P. Przedborski S. (2013). Pathogenesis of Parkinson's disease. Mov. Disord. 28, 24–30. 10.1002/mds.25032 22927094

[B91] Höglinger G. U. Barker R. A. Hagg T. Arias-Carrión O. Caldwell M. A. Hirsch E. C. (2012). Quantitative evaluation of the human subventricular zone. Brain. 135:e221, 1–4. 10.1093/brain/aws087 22539257

[B92] Höglinger G. U. Rizk P. Muriel M. P. Duyckaerts C. Oertel W. H. Caille I.. (2004). Dopamine depletion impairs precursor cell proliferation in Parkinson disease. Nat. Neurosci. 7, 726–735. 10.1038/nn1265 15195095

[B93] Hornykiewicz O. (1993). Parkinson's disease and the adaptive capacity of the nigrostriatal dopamine system: possible neurochemical mechanisms. Adv. Neurol. 60, 140–147. 8420131

[B94] Hu X. Zhang D. Pang H. Caudle W. M. Li Y. Gao H.. (2008). Macrophage antigen complex-1 mediates reactive microgliosis and progressive dopaminergic neurodegeneration in the MPTP model of Parkinson's disease. J. Immunol. 181, 7194–7204. 10.4049/jimmunol.181.10.7194 18981141 PMC2759089

[B95] Inestrosa N. C. Arenas E. (2010). Emerging role of Wnts in the adult nervous system. Nat. Rev. Neurosci. 11, 77–86. 10.1038/nrn2755 20010950

[B96] International Parkinson Disease Genomics Consortium Nalls M. A. Plagnol V. Hernandez D. G. Sharma M. Sheerin U. M.. (2011). Imputation of sequence variants for identification of genetic risks for Parkinson's disease: a meta-analysis of genome-wide association studies. Lancet 377, 641–649. 10.1016/S0140-6736(10)62345-8 21292315 PMC3696507

[B97] Jakubs K. Bonde S. Iosif R. E. Ekdahl C. T. Kokaia Z. Kopkaia M.. (2008). Inflammation regulates functional integration of neurons born in adult brain. J. Neurosci. 28, 12477–12488. 10.1523/JNEUROSCI.3240-08.2008 19020040 PMC6671710

[B98] Joksimovic M. Awatramani R. (2014). Wnt/β-catenin signaling in midbrain dopaminergic neuron specification and neurogenesis. J. Mol. Cell Biol. 6, 27–33. 10.1093/jmcb/mjt043 24287202

[B99] Jope R. S. Yuskaitis C. J. Beurel E. (2007). Glycogen synthase kinase-3 (GSK3): inflammation, diseases, and therapeutics. Neurochem. Res. 32, 577–595. 10.1007/s11064-006-9128-5 16944320 PMC1970866

[B100] Junn E. Lee K. W. Jeong B. S. Chan T. W. Im J. Y. Mouradian M. M. (2009). Repression of alpha-synuclein expression and toxicity by microRNA-7. Proc. Natl. Acad. Sci. U.S.A. 106, 13052–13057. 10.1073/pnas.0906277106 19628698 PMC2722353

[B101] Kalani M. Y. Cheshir S. H. Cord B. J. Bababeygy S. R. Vogel H. Weissman I. L.. (2008). Wnt-mediated self-renewal of neural stem/progenitor cells. Proc. Natl. Acad. Sci. U.S.A. 105, 16970–16975. 10.1073/pnas.0808616105 18957545 PMC2575225

[B102] Kannarkat G. T. Boss J. M. Tansey M. G. (2013). The role of innate and adaptive immunity in Parkinson's disease. J. Parkins. Dis. 3, 493–514. 10.3233/JPD-130250 24275605 PMC4102262

[B103] Kazanis I. (2009). The subependymal zone neurogenic niche: a beating heart in the centre of the brain: how plastic is adult neurogenesis? Opportunities for therapy and questions to be addressed. Brain 132, 2909–2921. 10.1093/brain/awp237 19773354 PMC2768664

[B104] Kilander M. B. Halleskog C. Schulte G. (2011). Recombinant WNTs differentially activate β-catenin-dependent and -independent signalling in mouse microglia-like cells. Acta Physiol. 203, 363–372. 10.1111/j.1748-1716.2011.02324.x 21557822

[B105] Kim B. Yang M. S. Choi D. Kim J. H. Kim H. S. Seol W.. (2012). Impaired inflammatory responses in murine Lrrk2-knockdown brain microglia. PLoS ONE 7:e34693. 10.1371/journal.pone.0034693 22496842 PMC3322140

[B106] Kim W. Y. Snider W. D. (2011). Functions of GSK-3 signaling in development of the nervous system. Front. Mol. Neurosci. 4:44. 10.3389/fnmol.2011.00044 22125510 PMC3221276

[B107] Kim W. Y. Wang X. Wu Y. Doble B. W. Patel S. Woodgett J. R.. (2009). GSK-3 is a master regulator of neural progenitor homeostasis. Nat. Neurosci. 12, 1390–1397. 10.1038/nn.2408 19801986 PMC5328673

[B108] King M. K. Pardo M. Cheng Y. Downey K. Jope R. S. Beurel E. (2013). Glycogen synthase kinase-3 inhibitors: rescuers of cognitive impairments. Pharmacol. Ther. 141, 1–12. 10.1016/j.pharmthera.2013.07.010 23916593 PMC3867580

[B109] Kokaia Z. Martino G. Schwartz M. Lindvall O. (2012). Cross-talk between neural stem cells and immune cells: the key to better brain repair? Nat. Neurosci. 15, 1078–1087. 10.1038/nn.3163 22837038

[B110] Koprich J. B. Reske-Nielsen C. Mithal P. Isacson O. (2008). Neuroinflammation mediated by IL-1beta increases susceptibility of dopamine neurons to degeneration in an animal model of Parkinson's disease. J. Neuroinflammation 5:8. 10.1186/1742-2094-5-8 18304357 PMC2292163

[B111] Kreutzberg G. W. (1996). Microglia: a sensor for pathological events in the CNS. Trends Neurosci. 19, 312–318. 10.1016/0166-2236(96)10049-7 8843599

[B112] Kuter K. Olech Ł. Głowacka U. (2017). Prolonged dysfunction of astrocytes and activation of microglia accelerate degeneration of dopaminergic neurons in the rat substantia nigra and block compensation of early motor dysfunction induced by 6-OHDA. Mol. Neurobiol. [Epub ahead of print]. 1–18. 10.1007/s12035-017-0529-z 28466266 PMC5842510

[B113] Kuwabara T. Hsieh J. Muotri A. Yeo G. Warashina M. Lie D. C.. (2009). Wnt-mediated activation of NeuroD1 and retro-elements during adult neurogenesis. Nat. Neurosci. 12, 1097–1105. 10.1038/nn.2360 19701198 PMC2764260

[B114] Langston J. W. (2006). The Parkinson's complex: Parkinsonism is just the tip of the iceberg. Ann. Neurol. 59, 591–596. 10.1002/ana.20834 16566021

[B115] Langston J. W. Forno L. S. Tetrud J. Reevers A. G. Kaplan J. A. Karluk D. (1999). Evidence of active nerve cell degeneration in the substantia nigra of humans years after 1-methyl-4-phenyl-1,2,3,6-tetrahydropyridine exposure. Ann. Neurol. 46, 598–605. 10514096 10.1002/1531-8249(199910)46:4<598::aid-ana7>3.0.co;2-f

[B116] Langston J. W. Sastry S. Chan P. Forno L. S. Bolin L. M. Di Monte D. A. (1998). Novel alpha-synuclein-immunoreactive proteins in brain samples from the Contursi kindred, Parkinson's, and Alzheimer's disease. Exp. Neurol. 154, 684–690. 10.1006/exnr.1998.6975 9878203

[B117] Lastres-Becker I. Ulusoy A. Innamorato N. G. Sahin G. Rabano A. Kirik D.. (2012). α-Synuclein expression and Nrf2-deficiency cooperate to aggravate protein aggregation, neuronal death and inflammation in early-stage Parkinson's disease. Hum. Mol. Genet. 21, 3173–3192. 10.1093/hmg/dds143 22513881

[B118] Latourelle J. C. Dimitriu A. Hadzi T. C. Beach T. G. Mayers R. H. Lewis P. (2012). Evaluation of Parkinson disease risk variants as expression-QTLs. PLoS ONE. 7:e46199. 10.1371/journal.pone.0046199 23071545 PMC3465315

[B119] Lee H. James W. S. Cowley S. A. (2017). LRRK2 in peripheral and central nervous system innate immunity: its link to Parkinson's disease. Biochem. Soc. Trans. 45, 131–139. 10.1042/BST20160262 28202666 PMC5652224

[B120] Lema Tomé C. M. Tyson T. Rey L. N. Grathwohl S. Britschgi M. Brundin P. (2012). Inflammation and alpha synuclein's prion-like behavior in Parkinson's disease-is there a link? Mol. Neurobiol. 29, 826–848. 10.1007/s12035-012-8267-8 PMC3589652 22544647

[B121] Lennington J. B. Pope S. Goodheart A. E. Drozdowicz L. Daniels S. B. Salamone J. D.. (2011). Midbrain dopamine neurons associated with reward processing innervate the neurogenic subventricular zone. J. Neurosci. 31, 13078–13087. 10.1523/JNEUROSCI.1197-11.2011 21917791 PMC3180856

[B122] L'Episcopo F. Drouin-Ouellet J. Tirolo C. Testa N. Caniglia S. Serapide M.-F.. (2016). GSK-3β-induced Tau pathology drives hippocampal neuronal cell death in Huntington's disease: involvement of astrocyte-neuron interactions. Cell Death Dis. 7:e2206. 10.1038/cddis.2016.104 27124580 PMC4855649

[B123] L'Episcopo F. Serapide M. F. Tirolo C. Testa N. Caniglia S. Morale M. C. (2011b). A Wnt1 regulated Frizzled-1/β-catenin signaling pathway as a candidate regulatory circuit controlling mesencephalic dopaminergic neuron-astrocyte crosstalk: therapeutical relevance for neuron survival and neuroprotection. Mol. Neurodeg. 13, 6–49. 10.1186/1750-1326-6-49 PMC3162575 21752258

[B124] L'Episcopo F. Tirolo C. Caniglia S. Testa N. Serra P. A. Impagnatiello F.. (2010b). Combining nitric oxide release with anti-inflammatory activity preserves nigrostriatal dopaminergic innervation and prevents motor impairment in a 1-methyl-4-phenyl-1,2,3,6-tetrahydropyridine model of Parkinson's disease. J. Neuroinflam. 7:83. 10.1186/1742-2094-7-83 21092260 PMC3000390

[B125] L'Episcopo F. Tirolo C. Testa N. Caniglia S. Morale M. C. Marchetti B. (2010a). Glia as a turning point in the therapeutic strategy of Parkinson's disease. CNS Neurol. Disord. 9, 349–372. 10.2174/187152710791292639 20438439

[B126] L'Episcopo F. Tirolo C. Testa N. Caniglia S. Morale M. C. Cossetti C.. (2011a). Reactive astrocytes and Wnt/β-catenin signaling link nigrostriatal injury to repair in 1-methyl-4-phenyl-1,2,3,6-tetrahydropyridine model of Parkinson's disease. Neurobiol. Dis. 41, 508–527. 10.1016/j.nbd.2010.10.023 21056667 PMC3558878

[B127] L'Episcopo F. Tirolo C. Testa N. Caniglia S. Morale M. C. Impagnatiello F.. (2011c). Switching the microglial harmful phenotype promotes lifelong restoration of subtantia nigra dopaminergic neurons from inflammatory neurodegeneration in aged mice. Rejuvenation Res. 14, 411–424. 10.1089/rej.2010.1134 21793734

[B128] L'Episcopo F. Tirolo C. Testa N. Caniglia S. Morale M. C. Serapide M. F. (2012). Plasticity of subventricular zone neuroprogenitors in MPTP (1-methyl-4-phenyl-1,2,3,6-tetrahydropyridine) mouse model of Parkinson's disease involves crosstalk between inflammatory and Wnt/β-catenin signaling pathways: functional consequences for neuroprotection and repair. J. Neurosci. 32, 2062–2085. 10.1523/JNEUROSCI.5259-11.2012 22323720 PMC3556384

[B129] L'Episcopo F. Tirolo C. Testa N. Caniglia S. Morale M. C. Impagnatiello F. (2013). Aging-induced Nrf2-ARE pathway disruption in the subventricular zone (SVZ) drives neurogenic impairment in parkinsonian mice via PI3K-Wnt/β-catenin dysregulation. J. Neurosci. 33, 1462–1485. 10.1523/JNEUROSCI.3206-12.2013 23345222 PMC3564519

[B130] L'Episcopo F. Tirolo C. Testa N. Caniglia S. Serapide M. F. Pluchino S. (2014a). Wnt/β-catenin signaling is required to rescue midbrain dopaminergic progenitors and restore nigrostriatal plasticity in ageing mouse model of Parkinson's disease. Stem Cells 32, 2147–2163. 10.1002/stem.1708 24648001 PMC4106883

[B131] L'Episcopo F. Tirolo C. Caniglia S. Testa N. Morale M. C. Serapide M. F.. (2014b). Targeting Wnt signaling at the neuroimmune interface for dopaminergic neuroprotection/ repair in Parkinson's disease. J. Mol. Cell Biol. 6, 13–26. 10.1093/jmcb/mjt053 24431301 PMC4061726

[B132] Liddelow S. A. Guttenplan K. A. Clarke L. E. Bennett F. C. Bohlen C. J. Schirmer L.. (2017). Neurotoxic reactive astrocytes are induced by activated microglia. Nature 541, 481–487. 10.1038/nature21029 28099414 PMC5404890

[B133] Lim D. A. Alvarez-Buylla A. (1999). Interaction between astrocytes and adult subventricular zone precursors stimulates neurogenesis. *Proc. Natl. Acad. Sci*. U.S.A. 96, 7526–7531. 10.1073/pnas.96.13.7526 PMC22119 10377448

[B134] Liu G. Abraham E. (2013). MicroRNAs in immune response and macrophage polarization. Arterioscler. Thromb. Vasc. Biol. 33, 170–177. 10.1161/ATVBAHA.112.300068 23325473 PMC3549532

[B135] Louafi F. Martinez-Nunez R. T. Sanchez-Elsner T. (2010). MicroRNA-155 targets SMAD2 and modulates the response of macrophages to transforming growth factor-beta. J. Biol. Chem. 285, 41328–41336. 10.1074/jbc.M110.146852 21036908 PMC3009858

[B136] Maiese K. Faqi L. Chong Z. Z. Shang Y. C. (2008). The Wnt signalling pathway: aging gracefully as a protectionist? Pharmacol. Ther. 118, 58–81. 10.1016/j.pharmthera.2008.01.004 18313758 PMC2432088

[B137] Marchetti B. Abbracchio M. P. (2005). To be or not to be (inflammed) is that the question in anti-inflammatory drug therapy of neurodegenerative diseases? Trends Pharmacol. Sci. 26, 517–525. 10.1016/j.tips.2005.08.007 16126283

[B138] Marchetti B. Pluchino S. (2013). Wnt your brain be inflamed? Yes, it Wnt! Trends Mol. Med. 19, 144–156. 10.1016/j.molmed.2012.12.001 23312954 PMC3595301

[B139] Marchetti B. Kettenmann H. Streit W. J. (2005a). Glia-neuron crosstalk in neuroinflammation, neurodegeneration and neuroprotection. Brain Res. Rev. Special Issue 482, 129–489. 10.1016/j.brainresrev.2004.12.002

[B140] Marchetti B. L'Episcopo F. Tirolo C. Testa N. Caniglia S. Morale M. C. (2011). Vulnerability to Parkinson's disease: towards an unifying theory of disease etiology, in Encyclopedia of Environmental Health, ed Nriagu J. O. (Burlington, US: Elsevier), 5, 690–704.

[B141] Marchetti B. L'Episcopo F. Morale M. C. Tirolo C. Testa N. Caniglia S.. (2013). Uncovering novel actors in astrocyte-neuron crosstalk in Parkinson's disease: the Wnt/β-catenin signaling cascade as the common final pathway for neuroprotection and self-repair. Eur. J. Neurosci. 37, 1550–1563. 10.1111/ejn.12166 23461676 PMC3660182

[B142] Marchetti B. Morale M. C. Testa N. Tirolo C. Caniglia S. Amor S.. (2002). Stress, the immune system and vulnerability to degenerative disorders of the central nervous system in transgenic mice expressing glucocorticoid receptor antisense RNA. Brain Res. Rev. 37, 259–272. 10.1016/S0165-0173(01)00130-8 11744091

[B143] Marchetti B. Serra P. A. L'Episcopo F. Tirolo C. Caniglia S. Testa N.. (2005b). Hormones are key actors in gene x environment interactions programming the vulnerability to Parkinson's disease: Glia as a common final pathway. Ann. N. Y. Acad. Sci. 1057, 296–318. 10.1196/annals.1356.023 16399902

[B144] Marchetti B. Serra P. A. Tirolo C. L'Episcopo F. Caniglia S. Gennuso F.. (2005c). Glucocorticoid receptor-nitric oxide crosstalk and vulnerability to experimental Parkinsonism: pivotal role for glia-neuron interactions. Brain Res. Rev. 48, 302–321. 10.1016/j.brainresrev.2004.12.030 15850669

[B145] Martino G. Pluchino S. Bonfanti L. Schwartz M. (2011). Brain regeneration in physiology and pathology: the immune signature driving therapeutic plasticity of neural stem cells. Physiol. Rev. 91, 1281–1304. 10.1152/physrev.00032.2010 22013212 PMC3552310

[B146] McGeer P. L. McGeer E. G. (2008). Glial reactions in Parkinson's disease. Mov Disord. 23, 474–483. 10.1002/mds.21751 18044695

[B148] Moehle M. S. Webber P. J. Tse T. Sukar N. Standaert D. G. DeSilva T. M.. (2012). LRRK2 inhibition attenuates microglial inflammatory responses. J. Neurosci. 32, 1602–1611. 10.1523/JNEUROSCI.5601-11.2012 22302802 PMC3532034

[B149] Morale M. C. L'Episcopo F. Tirolo C. Giaquinta G. Caniglia S. Testa N.. (2008). Loss of Aromatase Cytochrome P450 function as a risk factor for Parkinson's disease? Brain Res. Rev. 57, 431–443. 10.1016/j.brainresrev.2007.10.011 18063054

[B150] Morale M. C. Serra P. A. L'Episcopo F. Tirolo C. Caniglia S. Testa N.. (2006). Estrogen, neuroinflammation and neuroprotection in Parkinson's disease: glia dictates resistance versus vulnerability to neurodegeneration. Neuroscience 138, 869–878. 10.1016/j.neuroscience.2005.07.060 16337092

[B151] Morale M. C. Serra P. Delogu M. R. Migheli R. Rocchitta G. Tirolo C.. (2004). Glucocorticoid receptor deficiency increases vulnerability of the nigrostriatal dopaminergic system: critical role of glial nitric oxide. FASEB J. 18, 164–166. 10.1096/fj.03-0501fje 14630699

[B152] More S. V. Kumar H. Kim I. S. Song S. Y. Choi D. K. (2013). Cellular and molecular mediators of neuroinflammation in the pathogenesis of Parkinson's disease. Mediat. Inflamm. 2013:952375. 10.1155/2013/952375 23935251 PMC3712244

[B153] Mosimann C. Hausmann G. Basler K. (2009). Beta-catenin hits chromatin: regulation of Wnt target gene activation. Nat. Rev. Mol. Cell Biol. 10, 276–286. 10.1038/nrm2654 19305417

[B154] Mouton P. R. Long J. R. Lei D. L. Howard V. Jucker M. Calhoiun M. E. (2002). Age and gender effects on microglia and astrocytes in brains of mice. Brain Res. N956, 30–35. 10.1016/S0006-8993(02)03475-3 12426043

[B155] Neumann J. Schaale K. Farhat K. Endermann T. Ulmer A. J. Ehlers S.. (2010). Frizzled1 is a marker of inflammatory macrophages, and its ligand Wnt3a is involved in reprogramming Mycobacterium tuberculosis-infected macrophages. FASEB J. 24, 4599–4612. 10.1096/fj.10-160994 20667980

[B156] Niehrs C. (2012). The complex world of WNT receptor signalling. Nat. Rev. Mol. Cell. Biol. 13, 767–779. 10.1038/nrm3470 23151663

[B157] Njie E. G. Boelen E. Stassen F. R. Stassen F. R. Steinbusch H. W. Borchelt D. R. (2012). *Ex vivo* cultures of microglia from young and agent rodent brain reveal age-related changes in microglial function. Neurobiol. Aging 33, 195e1–12. 10.1016/j.neurobiolaging.2010.05.008 PMC4162517 20580465

[B158] Obeso J. A. Stamelou M. Goetz C. G. Poewe W. Lang A. E. Weintraub D.. (2017). Past, present, and future of Parkinson's disease: a special essay on the 200th anniversary of the shaking palsy. Movem. Disord. 32, 1265–1310. 10.1002/mds.27115 28887905 PMC5685546

[B159] O'Keeffe G. O. Barker R. A. Caldwell M. A. (2009a). Dopaminergic modulation of neurogenesis in the subventricular zone of the adult brain. Cell Cycle 8, 2888–2894. 10.4161/cc.8.18.9512 19713754

[B160] O'Keeffe G. O. Tyers P. Aarsland D. Dalley J. W. Barker R. A. Caldwell M. A. (2009b). Dopamine induced proliferation of adult neural precursor cells in the mammalian subventricular zone is mediated through EGF. Proc. Natl. Acad. Sci. U.S.A. 106, 8754–8759. 10.1073/pnas.0803955106 19433789 PMC2689002

[B161] Okamoto M. Inoue K. Iwamura H. Terashima K. Soya H. Asashima M.. (2011). Reduction in paracrine Wnt3 factors during aging causes impaired adult neurogenesis. FASEB J. 25, 3570–3582. 10.1096/fj.11-184697 21746862

[B162] Olanow C. W. Schapira A. (2013). Therapeutic prospects for parkinson's disease. Ann Neurol. 74, 337–347. 10.1002/ana.24011 24038341

[B163] Olanow C. W. Shapira A. H. Agid Y. (2003). Neuroprotection for Parkinson's disease: prospects and promises. Ann. Neurol. 53 (Suppl. 3), S1–S2. 10.1002/ana.10566 12666093

[B164] Orr C. F. Rowe D. B. Mizuno Y. Mori H. Halliday G. M. (2005). A possible role for humoral immunity in the pathogenesis of Parkinson's disease. Brain 128, 2665–2674. 16219675 10.1093/brain/awh625

[B165] Ouchi Y. Yagi S. Yokokura M. Sakamoto M. (2009). Neuroinflammation in the living brain of Parkinson's disease. Parkins. Relat. Disord. 15, S200–S204. 10.1016/S1353-8020(09)70814-4 20082990

[B166] Peng J. Peng L. Stevenson F. F. Doctrow S. R. Andersen J. K. (2007). Iron and paraquat as synergistic environmental risk factors in sporadic Parkinson's disease accelerate age-related neurodegeneration. J. Neurosci. 27, 6914–6922. 10.1523/JNEUROSCI.1569-07.2007 17596439 PMC6672233

[B167] Pereira C. P. Bachli E. B. Schoedon G. (2009). The Wnt pathway:a macrophage effector molecule that triggers inflammation. Curr. Atheroscler. Rep. 11, 236–242. 10.1007/s11883-009-0036-4 19361356

[B168] Perry V. H. Teeling J. (2013). Microglia and macrophages of the central nervous system: the contribution of microglia priming and systemic inflammation to chronic neurodegeneration. Semin. Immunopathol. 35, 601–612. 10.1007/s00281-013-0382-8 23732506 PMC3742955

[B169] Petit-Paitel A. Brau F. Cazareth J. Chabry J. (2009). Involment of cytosolic and mitochondrial GSK-3beta in mitochondrial dysfunction and neuronal cell death of MPTP/Mpp+- treated neurons. PLoS ONE 4:e5491 10.1371/journal.pone.0005491 19430525 PMC2675062

[B170] Phukan S. Babu V. S. Kannoji A. Hariharan R. Balaji V. N. (2010). GSK3b: role in therapeutic landscape and development of modulators. Br. J. Pharmacol. 160, 1–19. 10.1111/j.1476-5381.2010.00661.x 20331603 PMC2860202

[B171] Pluchino S. Zanotti L. Rossi B. Brambilla E. Ottoboni L. Salani G.. (2005). Neurosphere-derived multipotent precursors promote neuroprotection by an immunomodulatory mechanism. Nature 436, 266–271. 10.1038/nature03889 16015332

[B172] Pluchino S. Muzio L. Imitola J. Deleidi M. Al faro-Cervello C. Salani G.. (2008). Persistent inflammation alters the function of the endogenous brain stem cell compartment. Brain 131, 2564–2578. 10.1093/brain/awn198 18757884 PMC2570715

[B173] Ponomarev E. D. Veremeyko T. Weiner H. L. (2013). MicroRNAs are universal regulators of differentiation, activation, and polarization of microglia and macrophages in normal and diseased CN. Glia 61, 91–103. 10.1002/glia.22363 22653784 PMC3434289

[B174] Pott Godoy M. C. Tarelli R. Ferrari C. C. Sarchi M. I. Pitossi F. J. (2008). Central and systemic IL-1 exacerbates neurodegeneration and motor symptoms in a model of Parkinson's disease. Brain 131, 1880–1894. 10.1093/brain/awn101 18504291 PMC2442423

[B175] Pradhan S. Andreasson K.. (2013). Commentary: progressive inflammation as a contributing factor to early development of Parkinson's disease. Exp. Neurol. 241, 148–155. 10.1016/j.expneurol.2012.12.008 23261765

[B176] Prajapati P. Sripada L. Singh K. Bhatelia K. Singh R. (2015). TNF-α regulates miRNA targeting mitochondrial complex-I and induces cell death in dopaminergic cells. Biochim Biophys Acta 1852, 451–461. 10.1016/j.bbadis.2014.11.019 25481834

[B177] Prakash N. Wurst W. (2006). Genetic networks controlling the development of midbrain dopaminergic neurons. J. Physiol. 575, 403–410. 10.1113/jphysiol.2006.113464 16825303 PMC1819467

[B178] Przedborski S. (2010). Inflammation and Parkinson's disease pathogenesis. Mov. Disord. 25, S55–S57. 10.1002/mds.22638 20187228

[B179] Radad K. Moldzio R. Al-Shraim M. Kranner B. Krewenka C. Rausch W. D. (2017). Recent advances on the role of neurogenesis in the adult brain: therapeutic potential in Parkinson's and Alzheimer's diseases. CNS Neurol. Disord. Drug Targets 16, 740–748. 10.2174/1871527316666170623094728 28641510

[B180] Roose J. Molenaar M. Peterson J. Hurenkamp J. Brantjes H. Moerer P.. (1998). The *Xenopus* Wnt effector XTcf-3 interacts with Groucho-related transcriptional repressors. Nature 395, 608–612. 10.1038/26989 9783587

[B181] Rueger M. A. Muesken S. Walberer M. Jantzen S. U. Schnakenburg K. Backes H.. (2012). Effects of minocycline on endogenous neural stem cells after experimental stroke. Neuroscience 215, 174–183. 10.1016/j.neuroscience.2012.04.036 22542871

[B182] Sakata H. Niizuma K. Yoshioka H. Kim G. S. Jung J. E. Katsu M.. (2012). Minocycline-preconditioned neural stem cells enhance neuroprotection after ischemic stroke in rats. J. Neurosci. 32, 3462–3473. 10.1523/JNEUROSCI.5686-11.2012 22399769 PMC3315362

[B183] Salinas P. C. (2012). Wnt signaling in the vertebrate central nervous system: from axon guidance to synaptic function. Cold Spring Harb. Perspect. Biol. 4:a008003. 10.1101/cshperspect.a008003 22300976 PMC3281574

[B184] Sanchez-Guajardo V. Barnum C. J. Tansey M. G. Romero-Ramos M. (2013). Neuroimmunological processes in Parkinson's disease and their relation to α-synuclein: microglia as the referee between neuronal processes and peripheral immunity. ASN Neuro. 5, 113–139. 10.1042/AN20120066 23506036 PMC3639751

[B185] Sancho R. M. Law B. M. Harvey K. (2009). Mutations in the LRRK2 Roc-COR tandem domain link Parkinson's disease to Wnt signalling pathway. Hum. Mol. Genet. 18, 3955–3968. 10.1093/hmg/ddp337 19625296 PMC2748899

[B186] Schaale K. Neumann J. Schneider D. Ehlers S. Reiling N. (2011). Wnt signaling in macrophages: augmenting and inhibiting mycobacteria-induced inflammatory responses. Eur. J. Cell Biol. 90, 553–559. 10.1016/j.ejcb.2010.11.004 21185106

[B187] Schiess M. (2003). Nonsteroidal anti-inflammatory drugs protect against Parkinson neurodegeneration: can an NSAID a day keep Parkinson disease away? Arc. Neurol. 60, 1043–1044. 10.1001/archneur.60.8.1043 12925357

[B188] Schwartz M. London A. Shecter R. (2009). Boosting T-cell immunity as a therapeutic approach for neurodegenerative conditions: the role of innate immunity. Neuroscience 158, 1133–1142. 10.1016/j.neuroscience.2008.12.013 19103265

[B189] Seib D. R. Corsini N. S. Ellwanger K. Plaas C. Mateos A. Pitzer C.. (2013). Loss of Dickkopf-1 restores neurogenesis in old age and counteracts cognitive decline. Cell Stem Cell 12, 204–214. 10.1016/j.stem.2012.11.010 23395445

[B190] Shruster A. Ben-Zur T. Melamed E. Offen D. (2012). Wnt signaling enhances neurogenesis and improves neurological function after focal ischemic injury. PLoS ONE 7:e40843 10.1371/journal.pone.0040843 22815838 PMC3398894

[B191] Sofroniew M. V. Vinters H. V. (2010). Astrocytes: biology and pathology. Acta Neuropathol. 119, 7–35. 10.1007/s00401-009-0619-8 20012068 PMC2799634

[B192] Soifer H. S. Rossi J. J. Saetrom P. (2007). MicroRNAs in disease and potential therapeutic applications. Mol. Ther. 15, 2070–2079. 10.1038/sj.mt.6300311 17878899

[B193] Sonntag K. C. (2010). MicroRNAs and deregulated gene expression networks in neurodegeneration. Brain Res. 1338, 48–57. 10.1016/j.brainres.2010.03.106 20380815 PMC2883630

[B194] Sriram K. Matheson J. M. Benkovic S. A. Miller D. B. Luster M. I. O'Callaghan J. P. (2002). Mice deficient in TNF receptors are protected against dopaminergic neurotoxicity: implications for Parkinson's disease. FASEB J. 16, 1474–1476. 10.1096/fj.02-0216fje 12205053

[B195] Sriram K. Miller D. B. O'Callaghan J. P. (2006). Minocycline attenuates microglial activation but fails to mitigate striatal dopaminergic neurotoxicity: role of tumor necrosis factor-alpha. J. Neurochem. 96, 706–718. 10.1111/j.1471-4159.2005.03566.x 16405514

[B196] Staal F. J. Luis T. C. Tiemessen M. M. (2008). WNT signalling in the immune system: WNT is spreading its wings. Nat. Rev. Immunol. 8, 581–593. 10.1038/nri2360 18617885

[B197] Streit W. J. (2002). Microglia as neuroprotective, immunocompetent cells of the CN. Glia 40, 133–139. 10.1002/glia.10154 12379901

[B198] Streit W. J. (2010). Microglial activation and neuroinflammation in Alzheimer's disease: a critical examination of recent history. Front. Aging Neurosci. 2:22. 10.3389/fnagi.2010.00022 20577641 PMC2890154

[B199] Streit W. J. Mrak R. E. Griffin W. S. (2004). Microglia and neuroinflammation: a pathological perspective. J. Neuroinflammation. 1:14. 10.1186/1742-2094-1-14 15285801 PMC509427

[B200] Tansey M. G. Goldberg M. S. (2010). Neuroinflammation in Parkinson's disease: its role in neuronal death and implications for therapeutic intervention. Neurobiol. Dis. 37, 510–518. 10.1016/j.nbd.2009.11.004 19913097 PMC2823829

[B201] Taylor J. M. Main B. S. Crack P. J. (2013). Neuroinflammation and oxidative stress: co-conspirators in the pathology of Parkinson's disease. Neurochem. Int. 62, 803–819. 10.1016/j.neuint.2012.12.016 23291248

[B202] Teismann P. Tieu K. Choi D. K. Wu D. C. Naini A. Hunot S.. (2003). Cyclooxygenase-2 is instrumental in Parkinson's disease neurodegeneration. Proc. Natl. Acad. Sci. U.S.A. 100, 5473–5478. 10.1073/pnas.0837397100 12702778 PMC154369

[B203] Tepavcević V. Lazarini F. Alfaro-Cervello C. Kerninon C. Yoshikawa K. Garcia-Verdugo J. M.. (2011). Inflammation-induced subventricular zone dysfunction leads to olfactory deficits in a targeted mouse model of multiple sclerosis. J. Clin. Invest. 121, 4722–4734. 10.1172/JCI59145 22056384 PMC3226002

[B204] Thome A. D. Harms A. S. Volpicelli-Daley L. A. Standaert D. G. (2016). microRNA-155 regulates alpha-synuclein-induced inflammatory responses in models of Parkinson disease. J. Neurosci. 36, 2383–2390. 10.1523/JNEUROSCI.3900-15.2016 26911687 PMC4764660

[B205] Thored P. Heldmann U. Gomes-Leal W. Gisler R. Darsalia V. Taneera J.. (2009). Long-term accumulation of microglia with proneurogenic phenotype concomitant with persistent neurogenesis in adult subventricular zone after stroke. Glia 57, 835–849. 10.1002/glia.20810 19053043

[B206] Toledo E. M. Gyllborg D. Arenas E. (2017). Translation of WNT developmental programs into stem cell replacement strategies for the treatment of Parkinson's disease. Br. J. Pharmacol. 174, 4716–4724. 10.1111/bph.13871 28547771 PMC5727333

[B207] van Amerongen R. (2012). Alternative wnt pathways and receptors. Cold Spring Harb. Perspect. Biol. 4:a007914. 10.1101/cshperspect.a007914 22935904 PMC3475174

[B208] van Amerongen R. Mikels A. Nusse R. (2008). Alternative wnt signaling is initiated by distinct receptors. Sci. Signal. 1:re9. 10.1126/scisignal.135re9 18765832

[B209] Vegeto E. Belcredito S. Etteri S. Ghisletti S. Brusadelli A. Meda C.. (2003). Estrogen receptor-alpha mediates the brain antiinflammatory activity of estradiol. Proc. Natl. Acad. Sci. U.S.A. 100, 9614–9619. 10.1073/pnas.1531957100 12878732 PMC170966

[B210] Villeda S. A. Luo J. Mosher K. I. Zou B. Britschgi M. Bieri G.. (2011). The ageing systemic milieu negatively regulates neurogenesis and cognitive function. Nature 477, 90–94. 10.1038/nature10357 21886162 PMC3170097

[B211] Vukovic J. Colditz M. J. Blackmore D. G. Ruitenberg M. J. Bartlett P. F. (2012). Microglia modulate hippocampal neural precursor activity in response to exercise and aging. J. Neurosci. 32, 6435–6443. 10.1523/JNEUROSCI.5925-11.2012 22573666 PMC6621117

[B212] Wadhwa M. Prabhakar A. Ray K. Roy K. Kumari P. Jha P. K.. (2017). Inhibiting the microglia activation improves the spatial memory and adult neurogenesis in rat hippocampus during 48 h of sleep deprivation. J. Neuroinflammation. 14:222. 10.1186/s12974-017-0998-z 29141671 PMC5688670

[B213] Wallenquist U. Holmqvist K. Hånell A. Marklund N. Hillered L. Fosberg-Nilsson K. (2012). Ibuprofen attenuates the inflammatory response and allows formation of migratory neuroblasts from grafted stem cells after traumatic brain injury. Restorative Neurol. Neurosci. 30, 9–19. 10.3233/RNN-2011-0606 22377906

[B214] Wang S. Chong Z. Z. Shang Y. C. Maiese K. (2012). Wnt1 inducible signaling pathway protein 1 (WISP1) blocks neurodegeneration through phosphoinositide 3 kinase/Akt1 and apoptotic mitochondrial signaling involving Bad, Bax, Bim, and Bcl-xL. Curr. Neurovasc. Res. 9, 20–31. 10.2174/156720212799297137 22272766 PMC3274638

[B215] Wang W. Yang Y. Ying C. Li W. Ruan H. Zhu X.. (2007). Inhibition of glycogen synthase kinase-3β protects dopaminergic neurons from MPTP toxicity. Neuropharmacology 52, 1678–1684. 10.1016/j.neuropharm.2007.03.017 17517424

[B216] Wang X. J. Zhang S. Yan Z. Q. Zhao Y. X. Zhou H. Y. Wang Y.. (2011). Impaired CD200-CD200R-mediated microglia silencing enhances midbrain dopaminergic neurodegeneration: roles of aging, superoxide, NADPH oxidase, and p38 MAPK. Free Radic Biol. Med. 50, 1094–1106. 10.1016/j.freeradbiomed.2011.01.032 21295135

[B217] Wei L. Sun C. Lei M. Li G. Yi L. Luo F.. (2012). Activation of Wnt/β-catenin pathway by exogenous Wnt1 protects SH-SY5Y cells against 6-hydroxydopamine toxicity. J. Mol. Neurosci. 49, 105–115. 10.1007/s12031-012-9900-8 23065334

[B218] Whitton P. S. (2007). Inflammation as a causative factor in the aetiology of Parkinson's disease. Br. J. Pharmacol. 150, 963–976. 10.1038/sj.bjp.0707167 17339843 PMC2013918

[B219] Whitton P. S. (2010). Neuroinflammation and the prospects for anti-inflammatory treatment of Parkinson's disease. Curr. Opin. Investig. Drugs 11, 788–794. 20571974

[B220] Willert K. Nusse R. (2012). Wnt proteins. Cold Spring Harb. Perspect. Biol. 4:a007864. 10.1101/cshperspect.a007864 22952392 PMC3428774

[B221] Winner B. Winkler J. (2015). Adult neurogenesis in neurodegenerative diseases. Cold Spring Harb. Perspect. Biol. 7:a021287. 10.1101/cshperspect.a021287 25833845 PMC4382734

[B222] Winner B. Desplats P. Hagl C. Klucken J. Aigner R. Ploetz S.. (2009). Dopamine receptor activation promotes adult neurogenesis in an acute Parkinson model. Exp. Neurol. 219, 543–552. 10.1016/j.expneurol.2009.07.013 19619535 PMC5038985

[B223] Wurst W. Prakash N. (2014). Wnt-1 regulated genetic networks in midbrain dopaminergic neuron development. J. Mol. Cell Biol. 6, 34–41. 10.1093/jmcb/mjt046 24326514

[B224] Yang L. Tucker D. Dong Y. Wu C. Lu Y. Li Y.. (2017). Photobiomodulation therapy promotes neurogenesis by improving post-stroke local microenvironment and stimulating neuroprogenitor cells. Exp. Neurol. 299(Pt A):86–96. 10.1016/j.expneurol.2017.10.013 29056360 PMC5723531

[B225] Yang Y. Yang L. Liang X. Zhu G. (2015). MicroRNA-155 promotes atherosclerosis inflammation via targeting SOCS1. Cell Physiol. Biochem. 36, 1371–1381. 10.1159/000430303 26159489

[B226] Zeng X. Huang H. Tamai K. Zhang X. Harada Y. Yokota C.. (2008). Initiation of Wnt signaling: control of Wnt coreceptor Lrp6 phosphorylation/activation via frizzled, dishevelled and axin functions. Development 135, 367–375. 10.1242/dev.013540 18077588 PMC5328672

[B227] Zhang J. Götz S. Vogt Weisenhorn D. M. Simeone A. Wurst W. Prakash N.. (2015). A WNT1-regulated developmental gene cascade prevents dopaminergic neurodegeneration in adult En1(+/−) mice. Neurobiol. Dis. 82, 32–45. 10.1016/j.nbd.2015.05.015 26049140

[B228] Zhang J. Liu W. Wang Y. Zhao S. Chang N. (2017). miR-135b plays a neuroprotective role by targeting GSK3β in MPP(+)-intoxicated SH-SY5Y cells. Dis. Markers 2017:5806146. 10.1155/2017/5806146 28484287 PMC5412211

[B229] Zhang L. Yang X. Yang S. Zhang J. (2011). The Wnt/β-catenin signaling pathway in the adult neurogenesis. Eur. J. Neurosci. 33, 1–8. 10.1111/j.1460-9568.2010.7483.x 21073552

[B230] Zhang S. Wang X. J. Tian L. P. Pan J. Lu G. Q. Zhang Y. J.. (2011). CD200-CD200R dysfunction exacerbates microglial activation and dopaminergic neurodegeneration in a rat model of Parkinson's disease. J. Neuroinflammation 8:154. 10.1186/1742-2094-8-154 22053982 PMC3226566

[B231] Zhang W. Wang T. Pei Z. Miller D. S. Wu X. Blocl M. L.. (2005). Aggregated alpha synuclein activates microglia: a process leading to disease progression in Parkinson's disease. FASEB J. 19, 533–542. 10.1096/fj.04-2751com 15791003

[B232] Zhou Y. Lu M. Du R. H. Qiao C. Jiang C. Y. Zhang K.. (2016). MicroRNA-7 targets Nod-like receptor protein 3 inflammasome to modulate neuroinflammation in the pathogenesis of Parkinson's disease. Mol. Neurodegener. 11:28. 10.1186/s13024-016-0094-3 27084336 PMC4833896

